# Plasma metabolomic profiling identifies candidate biomarkers associated with sepsis diagnosis and mortality prediction

**DOI:** 10.3389/fimmu.2026.1931808

**Published:** 2026-10-14

**Authors:** Yanyan Zhu, Haocheng Wang, Sanjun Yi, Fuping You, Pingsen Zhao

**Affiliations:** 1 Department of Laboratory Medicine, Yuebei People’s Hospital Affiliated to Shantou University Medical College, Shaoguan, China; 2 Laboratory for Diagnosis of Clinical Microbiology and Infection, Yuebei People’s Hospital Affiliated to Shantou University Medical College, Shaoguan, China; 3 Research Center for Interdisciplinary and High-quality Innovative Development in Laboratory Medicine, Shaoguan, China; 4 Shaoguan Municipal Quality Control Center for Laboratory Medicine, Yuebei People’s Hospital Affiliated to Shantou University Medical College, Shaoguan, China; 5 Shaoguan Municipal Quality Control Center for Surveillance of Bacterial Resistance, Shaoguan, China; 6 Shaoguan Engineering Research Center for Research and Development of Molecular and Cellular Technology in Rapid Diagnosis of Infectious Diseases and Cancer, Shaoguan, China; 7 Institute of Systems Biomedicine, Department of Immunology, School of Basic Medical Sciences, Beijing Key Laboratory of Tumor Systems Biology, National Health Commission (NHC) Key Laboratory of Medical Immunology, Peking University Health Science Center, Beijing, China

**Keywords:** biomarker, diagnosis, LC-MS/MS, metabolomics, prognosis, sepsis

## Abstract

**Introduction:**

Sepsis is a life-threatening organ dysfunction caused by a dysregulated host response to infection; its early diagnosis and mortality prediction remain challenging, and conventional markers (CRP, PCT) have limited sensitivity and specificity. We aimed to characterize the plasma metabolome of sepsis and identify candidate metabolic biomarkers for diagnosis and 30-day mortality prediction.

**Methods:**

In this retrospective, single-center study, plasma from 30 healthy volunteers, 107 sepsis/septic shock patients and 19 non-septic ICU patients was analyzed by non-targeted LC-MS/MS and stratified into discovery (n=86) and replication (n=70) cohorts. Differential metabolites were defined by VIP > 1 and p < 0.05, with Benjamini–Hochberg q-values reported. Diagnostic and prognostic performance was assessed by ROC analysis and compared with CRP, PCT and the SOFA score; survival was analyzed by Kaplan–Meier with the log-rank test. A longitudinal subset was sampled on days 1, 3 and 5.

**Results:**

Multivariate analyses identified nine significantly dysregulated metabolites in sepsis patients versus both controls and non-septic ICU patients. Three—3-methoxyanthranilate, docosahexaenoic acid and N-acetyl-beta-alanine—showed particular significance, with alterations in amino-acid, carbohydrate and lipid metabolism, and were elevated in non-survivors. Longitudinal analysis revealed distinctive temporal patterns for 3-methoxyanthranilate and N-acetyl-beta-alanine. The combined panel showed diagnostic and prognostic performance that compared favorably with CRP, PCT and the SOFA score for sepsis differentiation and 30-day mortality.

**Discussion:**

3-methoxyanthranilate, docosahexaenoic acid and N-acetyl-beta-alanine are candidate metabolic biomarkers for sepsis diagnosis and prognosis. Given the modest, single-center sample, these exploratory, hypothesis-generating findings require confirmation in larger, multi-center cohorts and targeted metabolomic studies.

## Introduction

Sepsis represents a life-threatening pathophysiological condition characterized by a dysregulated host response to infection, manifesting as severe systemic inflammation and organ dysfunction. The progression of this complex syndrome frequently culminates in multiple organ dysfunction syndrome (MODS) and, in severe cases, septic shock, which is distinguished by profound impairment of tissue perfusion and oxygenation (1). These severe complications substantially contribute to the elevated morbidity and mortality rates associated with sepsis, establishing it as a predominant cause of mortality in intensive care units (ICUs) globally (2). Despite substantial research endeavors spanning several decades, the development of effective therapeutic interventions for sepsis remains challenging (3, 4). This clinical challenge is further exacerbated by the increasing prevalence of antimicrobial resistance and the decelerated pace of novel antibiotic development, emphasizing the critical necessity for innovative diagnostic and therapeutic strategies (5, 6). Contemporary diagnostic methodologies for sepsis, including established biomarkers such as C-reactive protein (CRP) and procalcitonin (PCT), demonstrate limited sensitivity and specificity, frequently resulting in delayed or inaccurate diagnoses. This diagnostic limitation underscores the imperative need for novel approaches in both diagnostic and therapeutic domains. Although no single metabolite biomarker or panel has yet entered routine clinical practice, prior metabolomic studies have begun to address this need. Recent work underscores both the promise of metabolomics for biomarker discovery in critically ill and septic patients and the translational challenges that remain (7–9).

Recent technological advances in *omics* methodologies, particularly metabolomics, present significant opportunities for expanding our understanding of sepsis pathophysiology and identifying novel diagnostic and prognostic biomarkers. Metabolomics, which encompasses the systematic analysis of small-molecule metabolites in biological specimens, has experienced substantial advancement through the rapid evolution of nuclear magnetic resonance (NMR) and mass spectrometry (MS) technologies (10–12). These sophisticated analytical platforms facilitate detailed metabolic profiling of pathological conditions, thereby providing crucial insights into disease mechanisms and potential therapeutic targets (13–16).

Notable contributions include the work of Ahn et al., who identified perturbations in plasma lysophosphatidylcholine (LPC)-related metabolic parameters within a murine sepsis model, elucidating the mechanisms underlying observed LPC level reductions during sepsis progression (17). Additionally, Chen et al. employed metabolomic analysis to distinguish between sepsis and systemic inflammatory response syndrome (SIRS), revealing crucial metabolic signatures that could facilitate early sepsis diagnosis (18). However, a significant research gap remains regarding the application of metabolomics for clinical diagnosis prediction and mortality risk assessment in sepsis patients. This knowledge deficit emphasizes the critical necessity for comprehensive clinical metabolomics investigations to elucidate the metabolic pathways implicated in sepsis pathogenesis and identify potential biomarkers for early diagnosis and prognosis.

## Materials and methods

### Study design and patient population

This retrospective observational study was conducted from July 2022 to March 2023 at Yuebei People’s Hospital Affiliated to Shantou University Medical College, China, encompassing 156 blood specimens obtained from three distinct cohorts: patients diagnosed with sepsis and septic shock (collectively designated as sepsis patients), ICU non-sepsis patients, and healthy control subjects. The study received approval from the Clinical Research Ethics Committee of Yuebei People’s Hospital Affiliated to Shantou University Medical College, China (Approval No: YBEC-KY(2021)-110), and all patients or their legal representatives have signed informed consent. Baseline (day-1) specimens for the discovery and replication cohorts were collected within 24 h of ICU admission, before or at the initiation of therapeutic interventions. For the longitudinal analysis, the day-3 and day-5 specimens were necessarily obtained during ongoing treatment; this is acknowledged as a limitation, as evolving therapy may contribute to the observed temporal metabolite changes.

The inclusion criteria for sepsis patients were (1): Confirmation of sepsis diagnosis based on Sequential Organ Failure Assessment (SOFA) score elevation of ≥ 2 points in response to infection, in accordance with the Third International Consensus Definitions for Sepsis and Septic Shock (Sepsis-3) (2); Age ≥ 18 years; and (3) Availability of comprehensive clinical documentation. Exclusion criteria were (1): Secondary admission to the intensive care unit (2); Presence of acute cardiovascular or cerebrovascular events, malignant neoplasms, or other terminal conditions (3); Pregnancy or lactation status (4); Documentation of autoimmune disorders requiring immunosuppressive or corticosteroid therapy (5); Mortality within 24 hours of study enrollment.

In addition to the general criteria, patients classified with septic shock were required to fulfill additional specific criteria (1): Documentation of persistent hypotension requiring vasopressor therapy to maintain mean arterial pressure ≥65 mmHg; and (2) Serum lactate concentrations >2 mmol/L (18 mg/dL). Therapeutic interventions for sepsis patients were implemented in strict accordance with international clinical practice guidelines. The inclusion criteria for ICU non-sepsis patients were (1): Requiring ICU admission for treatment, with no diagnosis of sepsis during the hospitalization (2). Age ≥ 18 years (3); Availability of comprehensive clinical documentation. The detailed clinical characteristics of subjects in both discovery and replication cohorts are presented in **Table 1**. To address the potential influence of organ dysfunction on the plasma metabolome, baseline blood urea, serum creatinine, estimated glomerular filtration rate (eGFR), and liver function tests (alanine aminotransferase, aspartate aminotransferase, and total bilirubin) were recorded for all participants, and the proportion of patients meeting KDIGO acute kidney injury (AKI) criteria, including Stage 3, is reported in **Table 1**.

**Table 1:** Clinical characteristics of subjects in the discovery and replication cohorts.

Discovery cohort							
	Clinical data by disease type				Clinical data by survival status		
Characteristic	Healthy (n=20)	Non-sepsis (n=11)	Sepsis (n=55)	*p value*	Survivor (n=34)	Death (n=21)	*p value*
Age (years)	52.0 (29.5–58.0)	55.0 (44.0–65.0)	69.0 (54.0–77.0)	0.001	61.5 (50.2–72.0)	73.0 (57.0–79.0)	0.059
SOFA score	0.0 (0.0–0.0)	0.0 (0.0–0.0)	7.0 (4.0–9.0)	<0.001	6.0 (3.2–8.0)	8.0 (7.0–11.0)	0.008
Sex, male, n (%)	9 (45)	9 (82)	35 (64)		22 (65)	13 (62)	
Comorbidities, n (%)							
Cardiovascular disease	0 (0)	0 (0)	9 (16)	0.059	5 (15)	4 (19)	0.719
Hypertension	0 (0)	0 (0)	17 (31)	0.003	9 (26)	8 (38)	0.386
Diabetes	4 (20)	2 (18)	13 (24)	0.894	8 (24)	5 (24)	1.000
COPD	0 (0)	0 (0)	1 (2)	0.752	0 (0)	1 (5)	0.382
Chronic kidney disease	0 (0)	0 (0)	11 (20)	0.029	4 (12)	7 (33)	0.082
Liver disease	0 (0)	0 (0)	13 (24)	0.013	6 (18)	7 (33)	0.208
Inflammatory and infection markers							
White blood cell count (×10^9^/L)	6.4 (5.3–8.2)	10.2 (7.3–13.8)	11.7 (7.8–18.6)	<0.001	12.9 (9.7–19.6)	10.0 (7.3–14.6)	0.058
C-reactive protein (mg/dL)	0.1 (0.0–0.1)	2.6 (0.9–14.4)	16.3 (8.3–23.4)	<0.001	18.2 (8.3–26.1)	15.0 (10.3–19.3)	0.662
Procalcitonin (ng/mL)	0.1 (0.0–0.1)	0.4 (0.1–2.3)	39.4 (11.1–100.0)	<0.001	40.7 (15.8–96.3)	32.0 (8.4–100.0)	0.706
Renal function							
Urea/BUN (mmol/L)	5.8 (4.5–6.2)	6.6 (5.2–6.8)	10.5 (7.3–14.8)	<0.001	9.2 (7.0–11.7)	12.0 (10.1–19.6)	0.012
Creatinine (µmol/L)	84.7 (65.6–94.9)	119.0 (78.5–127.5)	171.6 (107.9–253.0)	<0.001	138.5 (100.4–214.3)	242.6 (164.4–354.9)	0.011
eGFR (mL/min/1.73 m^2^)	88.6 (74.8–101.6)	63.3 (57.8–103.8)	36.1 (21.6–57.5)	<0.001	44.4 (26.9–65.2)	24.3 (13.1–40.9)	0.007
Hepatic function							
ALT (U/L)	15.0 (11.7–18.0)	28.9 (21.5–78.8)	41.3 (20.0–87.6)	<0.001	39.5 (18.1–67.7)	61.4 (21.6–106.7)	0.194
AST (U/L)	20.9 (18.1–22.8)	36.2 (28.8–66.9)	91.7 (34.1–165.0)	<0.001	74.0 (32.2–165.0)	96.5 (43.7–137.6)	0.525
Total bilirubin (µmol/L)	10.9 (7.4–14.3)	12.6 (10.0–28.2)	24.1 (12.4–59.8)	0.076	21.7 (12.2–46.8)	38.1 (16.8–62.3)	0.287
Acute kidney injury							
AKI stage 3, n (%)	–	–	10 (18)		4 (12)	6 (29)	
Outcome							
30-day mortality, n (%)	–	–	21 (38)		–	–	
Replication cohort							
	Clinical data by disease type				Clinical data by survival status		
Characteristic	Healthy (n=10)	Non-sepsis (n=8)	Sepsis (n=52)	*p value*	Survivor (n=35)	Death (n=17)	*p value*
Age (years)	45.5 (34.2–61.5)	48.5 (41.0–53.2)	64.0 (57.8–74.2)	0.001	64.0 (57.5–72.5)	72.0 (60.0–78.0)	0.287
SOFA score	0.0 (0.0–0.0)	1.5 (0.8–2.2)	5.0 (3.0–6.0)	<0.001	4.0 (3.0–6.0)	5.0 (5.0–6.0)	0.052
Sex, male, n (%)	5 (50)	6 (75)	27 (52)		16 (46)	11 (65)	
Comorbidities, n (%)							
Cardiovascular disease	0 (0)	0 (0)	16 (31)	0.028	9 (26)	7 (41)	0.340
Hypertension	0 (0)	0 (0)	19 (37)	0.011	13 (37)	6 (35)	1.000
Diabetes	0 (0)	2 (25)	13 (25)	0.204	10 (29)	3 (18)	0.506
COPD	0 (0)	0 (0)	1 (2)	0.839	1 (3)	0 (0)	1.000
Chronic kidney disease	0 (0)	0 (0)	17 (33)	0.019	13 (38)	4 (24)	0.358
Liver disease	0 (0)	0 (0)	9 (17)	0.167	6 (17)	3 (18)	1.000
Inflammatory and infection markers							
White blood cell count (×10^9^/L)	7.0 (6.6–8.1)	10.3 (9.6–10.9)	14.8 (9.8–19.9)	0.002	16.3 (11.4–24.8)	10.4 (7.8–16.4)	0.018
C-reactive protein (mg/dL)	0.1 (0.1–0.2)	11.2 (3.9–32.2)	25.5 (13.9–37.1)	<0.001	23.9 (11.9–30.2)	25.6 (20.8–38.2)	0.524
Procalcitonin (ng/mL)	0.0 (0.0–0.0)	5.9 (0.2–11.3)	23.6 (4.5–79.6)	<0.001	25.4 (5.2–98.2)	9.7 (4.8–39.7)	0.279
Renal function							
Urea/BUN (mmol/L)	4.8 (4.2–5.0)	9.2 (9.1–10.1)	11.6 (8.1–16.3)	<0.001	11.4 (8.3–15.9)	12.0 (7.8–17.7)	0.374
Creatinine (µmol/L)	73.6 (63.5–80.7)	127.8 (97.3–131.5)	132.3 (102.1–243.1)	0.002	131.8 (107.1–261.4)	132.3 (92.7–226.0)	0.742
eGFR (mL/min/1.73 m^2^)	107.2 (93.5–117.7)	58.9 (50.9–83.3)	44.0 (23.6–64.9)	<0.001	40.6 (20.7–61.8)	48.5 (29.0–68.9)	0.516
Hepatic function							
ALT (U/L)	14.2 (10.7–19.2)	29.3 (18.3–120.5)	24.3 (16.6–46.8)	0.025	20.9 (16.6–40.4)	31.7 (23.5–55.4)	0.334
AST (U/L)	19.8 (17.7–23.0)	43.0 (25.7–208.1)	40.1 (23.6–81.2)	0.013	36.8 (19.6–65.1)	72.0 (34.9–84.1)	0.146
Total bilirubin (µmol/L)	13.8 (10.7–15.3)	14.2 (8.3–21.5)	14.9 (9.9–34.2)	0.685	15.1 (11.4–32.2)	13.8 (8.7–37.3)	0.782
Acute kidney injury							
AKI stage 3, n (%)	–	–	7 (14)		5 (15)	2 (12)	
Outcome							
30-day mortality, n (%)	–	–	17 (33)		–	–	

The study design schema is depicted in **Figure 1**. The discovery panel encompassed: 21 patients with sepsis, 34 patients with septic shock, 11 ICU non-sepsis patients, and 20 healthy control subjects. The replication panel consisted of: 27 patients with sepsis, 25 patients with septic shock, 8 ICU non-sepsis patients, and 10 healthy control subjects. For longitudinal analysis, 23 patients diagnosed with sepsis and septic shock from the replication cohort were selected, comprising 11 survivors and 12 non-survivors (mortality within 30 days of enrollment). Participants were randomly assigned to the discovery and replication panels. Healthy control subjects were recruited from individuals attending routine health examinations during the same period and were free of acute illness, active infection, or known chronic inflammatory or immune disorders.

**Figure 1:**
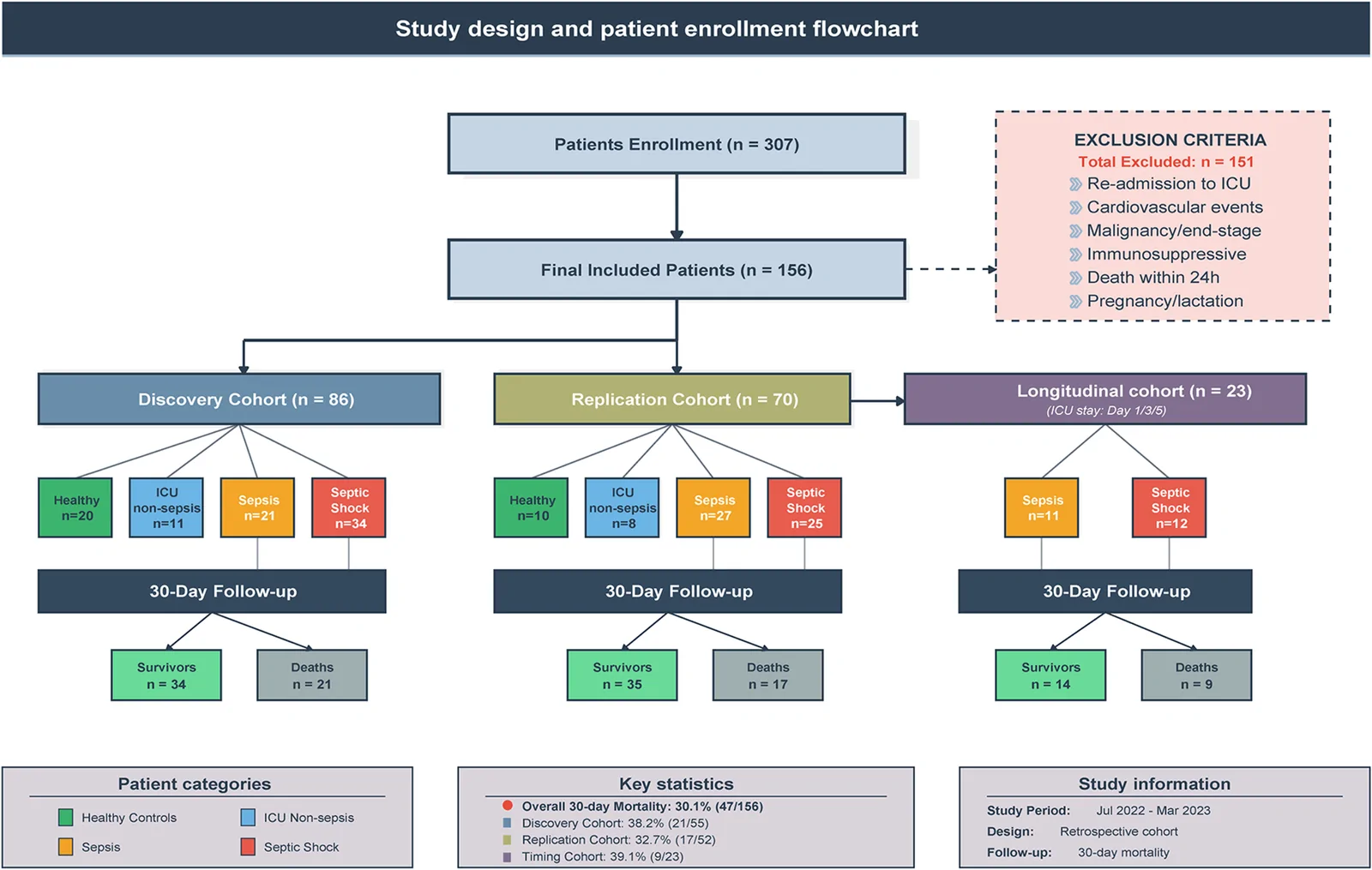
Flowchart and study design. Flowchart of individuals enrollment and study design in total patients with sepsis and subgroup analysis between survivors and deaths in sepsis, including timing analysis.

### Blood sample processing

Peripheral blood was collected into lithium-heparin tubes, consistent across all participants, and plasma was separated and stored at −80 °C until analysis. Frozen blood samples were thawed under controlled conditions at 4 °C and subjected to vortex mixing for 60 seconds to ensure complete homogenization. Precise aliquots were quantitatively transferred into 2 mL centrifuge tubes, followed by the addition of 400 µL pre-chilled methanol (-20 °C). The mixture was further homogenized by vortexing for 60 seconds and subsequently centrifuged at 12,000 rpm for 10 minutes at 4 °C. The resultant supernatant was carefully aspirated and transferred to fresh 2 mL centrifuge tubes, followed by vacuum concentration to complete dryness. The dried extracts were reconstituted in 150 µL of a solution comprising 80% methanol in water (v/v) containing 2-chloro-L-phenylalanine (4 ppm) as an internal standard, maintained at 4 °C. Prior to LC-MS analysis, the reconstituted samples were filtered through 0.22 µm membrane filters, and the filtrates were collected in autosampler vials.

### Liquid chromatography conditions

LC-MS grade methanol (MeOH) and acetonitrile (ACN) were procured from Fisher Scientific (Loughborough, UK). 2-Amino-3-(2-chloro-phenyl)-propionic acid was sourced from Aladdin (Shanghai, China), formic acid from TCI (Shanghai, China), and ammonium formate from Sigma-Aldrich (Shanghai, China). Ultrapure water was generated using a Milli-Q water purification system (Millipore, Bedford, USA). All reagents were of analytical grade and used as received.

Chromatographic separation was performed on a Vanquish UHPLC System (Thermo Fisher Scientific, USA) equipped with an ACQUITY UPLC^®^ HSS T3 column (2.1 × 100 mm, 1.8 µm particle size; Waters, Milford, MA, USA). The column temperature was maintained at 40 °C, with a flow rate of 0.3 mL/min and an injection volume of 2 µL. For positive electrospray ionization mode (ESI^+^), the mobile phase comprised (A2) 0.1% formic acid in water (v/v) and (B2) 0.1% formic acid in acetonitrile (v/v). The gradient elution program was as follows: 0–1 min, 8% B2; 1–8 min, linear increase from 8% to 98% B2; 8–10 min, maintained at 98% B2; 10-10.1 min, decrease from 98% to 8% B2; 10.1–12 min, re-equilibration at 8% B2 (16). For negative electrospray ionization mode (ESI^-^), the mobile phase consisted of (A3) 5 mM ammonium formate and (B3) acetonitrile. The gradient program was identical to that of the positive mode: 0–1 min, 8% B3; 1–8 min, linear increase from 8% to 98% B3; 8–10 min, maintained at 98% B3; 10-10.1 min, decrease from 98% to 8% B3; 10.1–12 min, re-equilibration at 8% B3 (19).

### Mass spectrometry conditions

Mass spectrometric analysis was conducted using an Orbitrap Exploris 120 mass spectrometer (Thermo Fisher Scientific, USA) interfaced with an electrospray ionization (ESI) source. The instrument was operated in Full MS-ddMS2 mode, enabling concurrent acquisition of MS1 and data-dependent MS/MS spectra. Operating parameters were optimized as follows: sheath gas pressure, 30 arbitrary units; auxiliary gas flow, 10 arbitrary units; spray voltage, 3.50 kV for positive mode and -2.50 kV for negative mode; capillary temperature, 325 °C. MS1 data were acquired over a mass range of m/z 100–1000 at a resolution of 60,000 FWHM. Data-dependent MS/MS acquisition incorporated four scans per duty cycle at 15,000 FWHM resolution, with normalized collision energy of 30% and automatic dynamic exclusion (20).

### Data processing

Raw mass spectrometric data were converted to mzXML format using MSConvert (ProteoWizard software package, v3.0.8789) and subsequently processed using XCMS (v3.12.0) (21) for feature detection, retention time alignment, and peak picking. Metabolite identification was performed by comparing accurate mass measurements (mass error <30 ppm) and MS/MS fragmentation patterns against multiple reference databases, including the Human Metabolome Database (HMDB), MassBank, LIPID MAPS, mzCloud, and the Kyoto Encyclopedia of Genes and Genomes (KEGG). Quality control-based robust LOESS signal correction (QC-RLSC) was implemented for data normalization to minimize systematic variations. For quality assurance, only metabolite features exhibiting relative standard deviations (RSDs) below 30% in quality control samples were retained for subsequent analysis. Sample acquisition was performed by the analytical service provider under a standardized untargeted-metabolomics workflow that incorporated pooled quality-control (QC) samples to monitor instrument stability throughout the run, with QC-based robust LOESS signal correction applied during data processing. Metabolite annotations were assigned at Metabolomics Standards Initiative (MSI) Level 2 (spectral-library match without authentic-standard confirmation); the full identification evidence for the nine panel metabolites—including measured m/z, mass error, adduct, retention time and database identifiers—is provided in **Supplementary Table 2**.

### Multivariate analysis

Multivariate statistical analyses were performed using the ropls package in R. Data matrices were preprocessed through mean-centering and scaling prior to analysis (22). Principal component analysis (PCA), orthogonal partial least squares discriminant analysis (OPLS-DA), and partial least squares discriminant analysis (PLS-DA) were employed to construct models. Metabolic profiles were visualized through score plots, with each point representing an individual sample. Loading plots and S-plots were concurrently generated to identify metabolites that significantly influenced sample clustering. To assess the risk of overfitting, permutation tests were conducted on all models. The descriptive performance of the models was evaluated using cumulative R^2^X (R^2^X(cum) = 1 indicates a perfect model) and R^2^Y (R^2^Y(cum) = 1 indicates a perfect model) values, while predictive performance was measured using cumulative Q^2^ (Q^2^(cum) = 1 indicates a perfect model) and permutation tests. Validation of the models ensured that permuted models did not accurately predict class distinctions, as evidenced by R^2^ and Q^2^ values at the Y-axis intercept that were lower than those of the non-permuted models. OPLS-DA was used to identify discriminant metabolites based on variable importance in projection (VIP) scores. Metabolites with *p* < 0.05 and VIP > 1 were considered differential at the screening stage. Given the exploratory, hypothesis-generating aim of this study and the use of a multivariate (OPLS-DA/VIP) filter, no formal false-discovery-rate (FDR) correction was applied at the screening stage; Benjamini–Hochberg-adjusted q-values are nonetheless provided for all differential metabolites in **Supplementary Table 3**. The sepsis-versus-healthy signatures were robust to FDR correction, whereas the sepsis-versus–non-septic-ICU comparison, particularly in the replication cohort, did not survive correction, consistent with the more subtle metabolic differences between critically ill groups and the modest sample size.

### Pathway analysis

Differentially abundant metabolites were subjected to comprehensive pathway analysis using MetaboAnalyst, integrating both enrichment and topology analyses. The identified metabolites were systematically mapped to metabolic pathways using KEGG reference database. Pathway significance was evaluated based on two complementary aspects (1): Assessment of metabolite over-representation within specific pathways (2); Evaluation of metabolite positions and their impact on pathway function. Pathway visualization and network analysis were performed using KEGG Mapper, enabling the contextualization of metabolic alterations within broader biological systems. This approach facilitated the identification of significantly perturbed metabolic pathways and their potential biological implications.

### Statistical analysis

The normality of continuous variables was assessed and, as most were non-normally distributed, continuous variables were expressed as median (interquartile range) and compared using the Mann–Whitney U test for two groups and the Kruskal–Wallis test (or one-way ANOVA where distributional assumptions were met) for three or more groups for three or more groups; categorical variables were expressed as counts (percentages) and compared using the χ^2^ test or Fisher’s exact test, as appropriate. Multivariate analyses (PCA, OPLS-DA and PLS-DA) were performed with the ropls package, with 200-fold permutation testing to assess model robustness. Metabolites with variable importance in projection (VIP) > 1 and *p* < 0.05 were considered differential, and Benjamini–Hochberg false-discovery-rate *q*-values are additionally reported. The discovery and replication cohorts were analyzed independently and in parallel, each undergoing the full pipeline separately; ROC analyses in the discovery cohort were performed before the cross-cohort selection step. The candidate panel was then defined as the intersection of metabolites showing significant, concordant differential abundance in both cohorts. Because this selection step used the replication cohort and the panel’s diagnostic and prognostic ROC analyses were subsequently evaluated within the replication cohort, those replication-cohort performance estimates are not fully independent of selection and may be optimistic. ROC analyses were performed with the pROC and multipleROC packages, and AUC with 95% confidence intervals; formal pairwise comparison of ROC curves (DeLong test), model calibration and internal validation were not performed. Survival was analyzed by the Kaplan–Meier method with the log-rank test (survival and survminer packages), dichotomising patients at the median metabolite level. All tests were two-sided and *p* < 0.05 was considered statistically significant. Analyses were performed in R (version 4.0.3; key packages: ropls v1.22.0, pROC v1.19.1, survival v3.8 and survminer v0.5.2); raw spectra were processed with XCMS and pathway analysis performed with MetaboAnalyst as described above.

## Results

### Comprehensive metabolomic profiling of sepsis in the discovery cohort

The overall study design is illustrated in **Figure 1**, which summarizes patient stratification, biospecimen collection, metabolomic profiling, and subsequent bioinformatics analyses. The analytical integrity was validated through rigorous quality control procedures. QC samples demonstrated high analytical reproducibility and consistent clustering patterns across both ionization modes, confirming the methodological robustness for both study cohorts (**Supplementary Figure 1A**). OPLS-DA modeling revealed clear metabolic distinctions between sepsis patients and healthy controls in both positive and negative ionization modes (**Supplementary Figure 1B**). Model validation through permutation testing demonstrated robust predictive capability, with R^2^ values exceeding Q^2^ values and negative Y-axis intercepts, confirming the absence of model overfitting (**Supplementary Figure 1C**).

To characterize the plasma metabolome of sepsis, we performed untargeted dual-polarity LC-MS/MS profiling in a discovery cohort comprising healthy controls (n = 20), non-septic ICU patients (n = 11), sepsis patients (n = 21) and septic-shock patients (n = 34); sepsis and septic shock were combined as a single sepsis group (SEP_SEPS, n = 55) for all analyses. Plasma was collected within 24h of ICU admission and analyzed in both ionization modes, followed by data processing, quality control and metabolite annotation (**Figure 2A**). In total, 4,941 metabolites were identified (2,869 in ESI^+^ and 2,072 in ESI^−^ mode; **Supplementary Figure 1D**). After quality-control filtering, 741 metabolites were quantified and retained for comparative analysis, of which 561 showed higher and 180 lower mean abundance in sepsis patients than in healthy controls when classified by direction of fold change alone. Unsupervised three-dimensional principal component analysis of the quantified metabolites revealed clear separation between sepsis patients and healthy controls in both ESI^+^ and ESI^−^ modes, indicating a substantially altered plasma metabolome in sepsis (**Figure 2B**).

**Figure 2:**
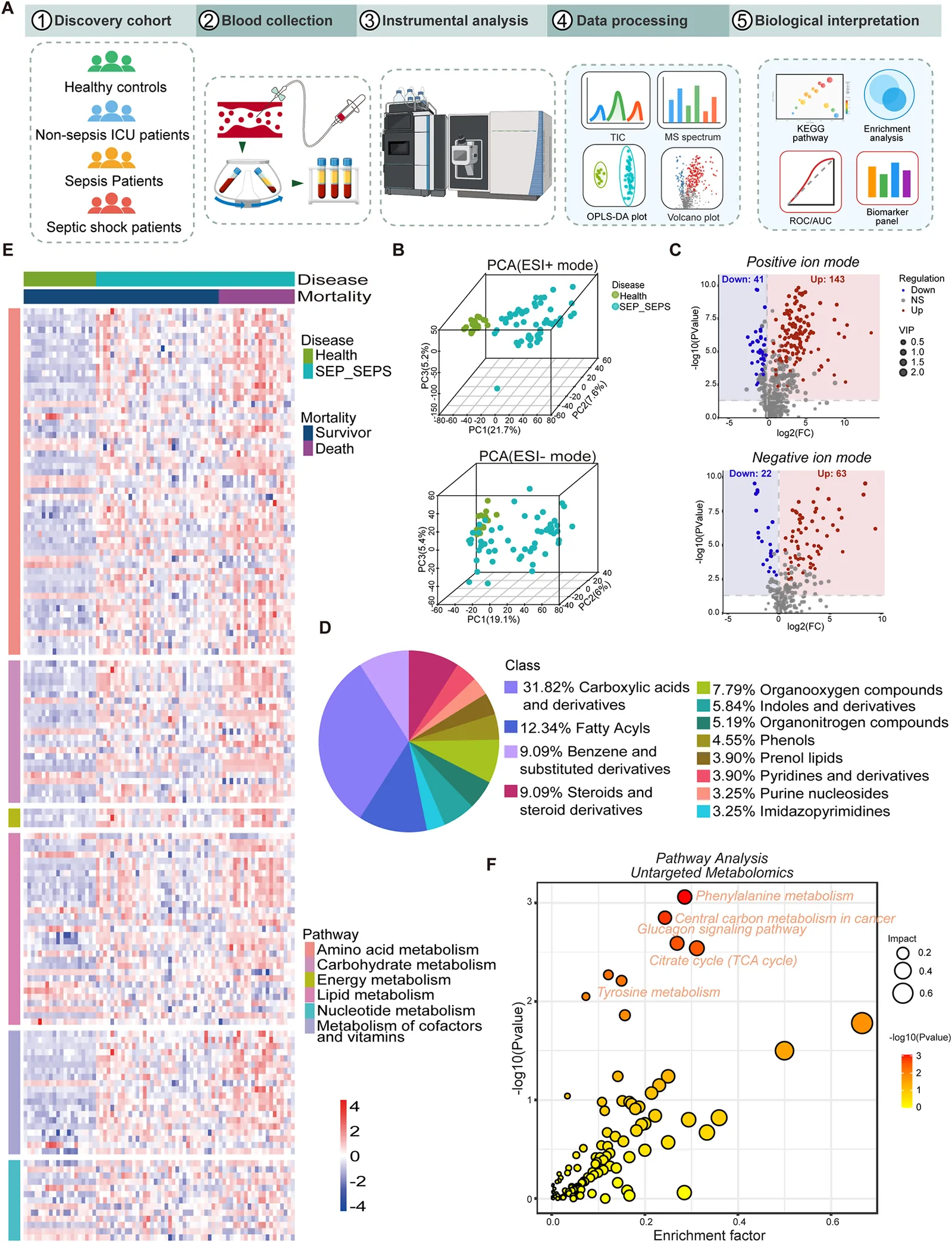
Metabolomics analysis identifies significant metabolic differences between sepsis patients and healthy controls. **(A)** Overview of study design and analytical workflow in discovery cohort. Flow diagram depicting patient stratification into sepsis, septic shock, ICU non-sepsis, and healthy control groups, followed by standardized blood sample collection, tandem mass spectrometry metabolomic analysis, computational data processing, and comprehensive bioinformatics evaluation. **(B)** 3D PCA showing the separation of patient samples between the sepsis group (blue) and the healthy control group (green), indicating distinct metabolic profiles. **(C)** Volcano plots depicting the statistical significance (log_10_ *p*-value) versus the magnitude of change (log_2_ fold change) for metabolites in the discovery group. Metabolites with adjusted *p*-values < 0.05 and |log2 fold change| > 1 are highlighted. **(D)** Pie chart showing the 12 most represented chemical classes among the differential metabolites (154 of the 213 classified metabolites); segment size reflects each class’s proportion within these 12 classes. **(E)** Hierarchical clustering heatmap of the 146 differential metabolites in the discovery cohort (sepsis versus healthy controls), annotated by KEGG metabolic pathway category. Rows represent metabolites and columns represent individual samples (20 healthy controls and 55 sepsis patients); color intensity indicates row-scaled relative abundance. **(F)** The scatter plot displays the enrichment factor (x-axis) versus −log_10_(*P*-value) (y-axis). Circle size corresponds to pathway impact, and color intensity reflects statistical significance (−log_10_(*P*-value)).

To identify metabolites that differed significantly between the two groups, we applied a combined threshold of *p* < 0.05 and VIP > 1. This yielded 269 significant differential metabolites (206 increased and 63 decreased in sepsis relative to healthy controls; **Supplementary Table 3**), comprising 143 increased and 41 decreased metabolites in ESI^+^ mode and 63 increased and 22 decreased in ESI^−^ mode (**Figure 2C**). We next characterized these 269 differential metabolites. Assignment to chemical classes showed that 213 could be categorized into 52 chemical classes, of which the 12 most represented classes (comprising 154 metabolites) are shown in **Figure 2D**. Carboxylic acids and their derivatives constituted the predominant class (49 metabolites; 31.82% of the top 12 classes), followed by fatty acyls (19; 12.34%), with benzene and substituted derivatives and steroids and steroid derivatives each accounting for 9.09% (14 metabolites each) (**Figure 2D**). To place these metabolites in a functional context, we mapped them to KEGG pathways. Of the 269 differential metabolites, 146 were mapped to KEGG pathways and grouped into six metabolic categories: amino-acid, carbohydrate, energy, lipid, nucleotide, and cofactor-and-vitamin metabolism. Hierarchical clustering of these 146 metabolites across the 20 healthy-control and 55 sepsis samples separated sepsis patients from healthy controls, tended to distinguish survivors from non-survivors, with most of these metabolites showing higher abundance in non-survivors (**Figure 2E**). Amino-acid metabolism contributed the largest number of mapped entries (n = 56), followed by lipid (n = 31), carbohydrate (n = 23), cofactor-and-vitamin (n = 20), nucleotide (n = 13) and energy metabolism (n = 3). KEGG pathway enrichment analysis of the differential metabolites highlighted 63 pathways across these six categories (**Figure 2F**). The most enriched pathways by nominal *p*-value were phenylalanine metabolism (11 metabolites; *p* = 0.0009), the citrate (TCA) cycle (6; *p* = 0.003), tyrosine metabolism (13; *p* = 0.005) and arginine biosynthesis (6; *p* = 0.006). These results should be interpreted as exploratory: no pathway remained significant after Benjamini–Hochberg correction (all FDR > 0.05), consistent with the modest sample size, so the enrichment findings are presented as hypothesis-generating rather than confirmatory.

To determine whether these alterations were specific to sepsis rather than to critical illness in general, we applied the same workflow to sepsis versus non-septic ICU patients. Quality-control assessment, OPLS-DA and permutation testing confirmed the validity and robustness of the models (**Supplementary Figures 2A–C**). Among these, 741 quantified metabolites were retained for differential analysis, of which 529 showed increased and 212 decreased abundance in sepsis patients relative to non-septic ICU patients (**Supplementary Figure 2D**), and three-dimensional PCA again clearly separated the two groups (**Supplementary Figure 3A**). Using the same threshold (*p* < 0.05, VIP > 1), 255 significant differential metabolites were identified (190 increased and 65 decreased in sepsis; **Supplementary Table 3**) (**Supplementary Figure 3B**). As in the sepsis-versus-healthy comparison, carboxylic acids and their derivatives were the predominant chemical class (33.33% of the top 12 classes; **Supplementary Figure 3C**), and both the six KEGG pathway categories and the most enriched pathways—led by phenylalanine metabolism and the citrate (TCA) cycle—closely paralleled those observed against healthy controls (**Supplementary Figures 3D–F**). This strong concordance across two independent comparators indicates that the observed alterations reflect sepsis-associated metabolic reprogramming rather than non-specific features of ICU admission.

### Identification of biomarker metabolites for sepsis diagnosis and prognosis

To identify potential diagnostic biomarker metabolites utilizing the discovery cohort data, we implemented pairwise comparative analyses to detect significantly differentiated metabolites between groups. Metabolite significance was stringently defined by VIP values exceeding 1.0 and *p*-values below 0.05. The comparative analysis between sepsis patients and healthy controls yielded 269 significantly differentiated metabolites. Similarly, the comparison between sepsis and ICU non-sepsis groups revealed 255 significantly differentiated metabolites, with 116 upregulated and 16 downregulated metabolites common to both comparative analyses relative to healthy controls (**Figure 3A**). These findings demonstrated that metabolite profiles effectively discriminated the sepsis cohort from both healthy controls and ICU non-sepsis patients (**Figure 3B**). To assess the diagnostic utility of the identified metabolites, we conducted ROC curve analyses. The top 20 differentially expressed metabolites were stratified into five hierarchical clusters, each comprising four metabolites, and their diagnostic performance was compared with established clinical markers, CRP and PCT. These metabolites showed numerically higher AUC than CRP (AUC = 0.78), highlighting their potential as candidate diagnostic biomarkers for sepsis (**Figure 3C**).

**Figure 3:**
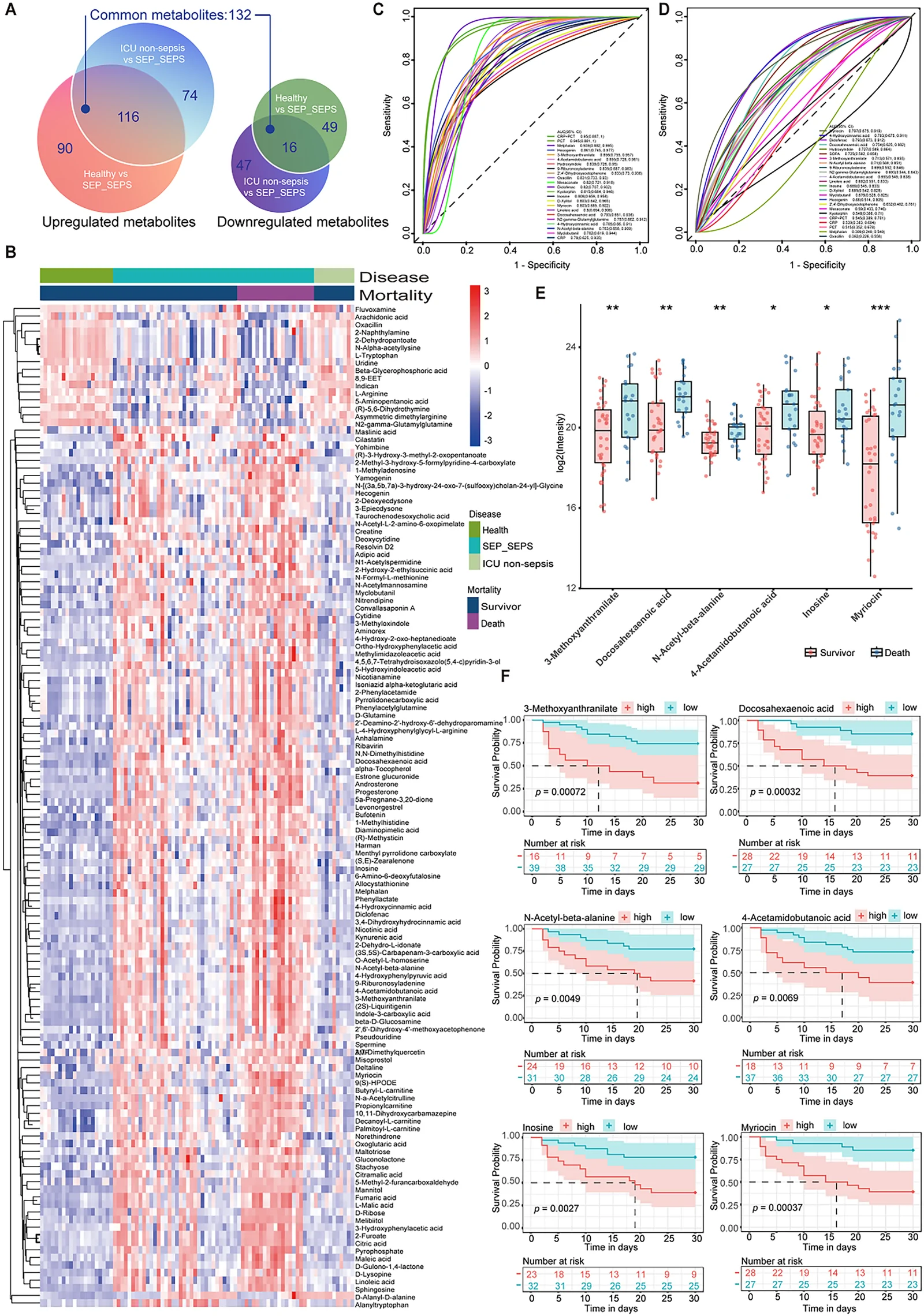
Screening and application of potential biomarkers in sepsis diagnosis and prognostic assessment. **(A)** Venn diagrams showing the overlap of significantly altered metabolites between comparisons. (Left) Upregulated metabolites in SEP_SEPS compared with healthy controls and ICU non-sepsis patients. (Right) Downregulated metabolites in SEP_SEPS compared with healthy controls and ICU non-sepsis patients. **(B)** Heatmap displaying the expression levels of shared differentially expressed metabolites among sepsis patients (blue), ICU non-sepsis patients (light green) and healthy controls (dark green), with rows representing metabolites and columns representing individual samples. Color intensity reflects the relative metabolite abundance, with higher levels in red and lower levels in green. **(C)** ROC curve assessing the diagnostic efficacy of categorized differentially expressed metabolites in distinguishing sepsis patients from healthy controls. The AUC represents the overall diagnostic accuracy. **(D)** ROC curves evaluating the predictive ability of categorized differentially expressed metabolites for mortality in sepsis patients. The AUC indicates the capacity of these metabolites to forecast the risk of death. **(E)** Bar graph presenting the differential expression levels of representative metabolites in relation to survival (red) and mortality (blue) among sepsis patients, with statistical significance indicated. **(F)** Kaplan-Meier survival plots illustrating the 30-day mortality outcomes associated with the representative metabolites, comparing survival rates between patient groups.

Subsequent analyses evaluated the prognostic potential of the 20 metabolites identified through hierarchical clustering for predicting 30-day mortality in sepsis patients. ROC curve analyses demonstrated that the majority of these metabolites showed numerically higher prognostic performance than conventional clinical markers, including CRP, PCT, and their combination (CRP+PCT). The AUC values for traditional markers were 0.545 (CRP+PCT), 0.539 (CRP), and 0.515 (PCT), with the identified metabolites demonstrating numerically higher predictive capability (**Figure 3D**). To further validate the prognostic utility of these metabolites, we conducted detailed analyses of six representative compounds: 3-methoxyanthranilate, docosahexaenoic acid, N-acetyl-beta-alanine, 4-acetamidobutanoic acid, inosine, and myriocin. Comparative analyses of expression levels between survival and mortality groups, coupled with correlation analyses against 30-day survival outcomes, revealed significantly elevated expression of all six metabolites in the mortality group. These metabolites demonstrated positive correlations with 30-day mortality, suggesting their potential value as prognostic indicators in sepsis patients (**Figures 3E, F**).

### Metabolic alterations are reproduced in a replication cohort

To validate these findings, we applied the same workflow to a replication cohort comprising healthy controls (n = 10), non-septic ICU patients (n = 8), sepsis patients (n = 27) and septic-shock patients (n = 25), with sepsis and septic shock combined as a single sepsis group (**Figure 4A**). The analytical reliability and reproducibility of the LC-MS/MS methodology were validated through QC sample analyses, which demonstrated consistent clustering in both ionization modes (**Supplementary Figure 4A**). OPLS-DA revealed distinct metabolic segregation between healthy controls and sepsis patients, corroborating the discriminatory power of the identified metabolic profiles (**Supplementary Figure 4B**). The statistical robustness of the OPLS-DA model was further substantiated through permutation testing (**Supplementary Figure 4C**). In total, 4,620 metabolites were identified (2,985 in ESI^+^ and 1,635 in ESI^−^ mode), of which 632 were quantified and retained for comparative analysis; classified by direction of fold change alone, 372 showed higher and 260 lower mean abundance in sepsis than in healthy controls (**Supplementary Figure 4D**). Three-dimensional PCA separated sepsis patients from healthy controls in both ionization modes (**Figure 4B**). Applying the same threshold (*p* < 0.05, VIP > 1) identified 220 significant differential metabolites (129 increased and 91 decreased in sepsis; **Figure 4C**; **Supplementary Table 3**). As in the discovery cohort, carboxylic acids and their derivatives were the predominant chemical class (40 metabolites; 29.63% of the top 12 classes), followed by benzene and substituted derivatives and organooxygen compounds (11.11% each) (**Figure 4D**). Mapping the differential metabolites to KEGG pathways, 131 metabolites were grouped into the same six metabolic categories and clustered to separate sepsis patients from healthy controls across the 10 healthy-control and 52 sepsis samples (**Figure 4E**). Pathway-level analysis (**Figure 4F**) highlighted shifts in amino-acid and central-carbon metabolism—including the TCA cycle, phenylalanine metabolism, alanine, aspartate and glutamate metabolism, and arginine biosynthesis—closely mirroring the pathways perturbed in the discovery cohort. Consistent with the discovery cohort, these enrichment results are presented as exploratory, given the modest sample size. Finally, we repeated the sepsis versus non-septic ICU comparison in the replication cohort. Quality control, OPLS-DA and permutation testing confirmed model validity (**Supplementary Figures 5A–C**), and three-dimensional PCA separated the two groups (**Supplementary Figure 6A**). 394 showed increased and 238 decreased abundance in sepsis relative to ICU non-sepsis (**Supplementary Figure 5D**). Using the same threshold (*p* < 0.05, VIP > 1), 100 significant differential metabolites were identified (72 increased and 28 decreased in sepsis; **Supplementary Figure 6B**; **Supplementary Table 3**). Consistent with all preceding comparisons, carboxylic acids and their derivatives were the predominant chemical class (18 metabolites; 31.58% of the top 12 classes; **Supplementary Figure 6C**), and the differential metabolites again mapped to the same broad metabolic categories—amino-acid, carbohydrate, lipid, nucleotide, energy, and cofactor-and-vitamin metabolism (**Supplementary Figures 6D–F**). Although this comparison yielded the fewest differential metabolites and no pathway retained significance after FDR correction, the recurrence of a carboxylic-acid– and amino-acid–dominated signature across two cohorts and two comparators supports its association with sepsis rather than with ICU admission per se. These results are exploratory and hypothesis-generating. Following identical analytical protocols (**Supplementary Figures 7A, B**), we identified 20 metabolites based on their diagnostic potential. These metabolites showed numerically higher discriminatory capability between sepsis patients and both healthy controls and non-sepsis ICU patients, achieving an AUC of 0.702, higher than conventional markers CRP and PCT (**Supplementary Figures 7C, D**). Detailed analysis of three selected metabolites—linoleic acid, lysophosphatidic acid (LysoPA 16:0), and neopterin—revealed their potential utility as prognostic biomarkers for sepsis. LysoPA (16:0) showed elevated expression levels in non-surviving patients, while linoleic acid and neopterin levels were diminished in this cohort (**Supplementary Figures 7E, F**).

**Figure 4:**
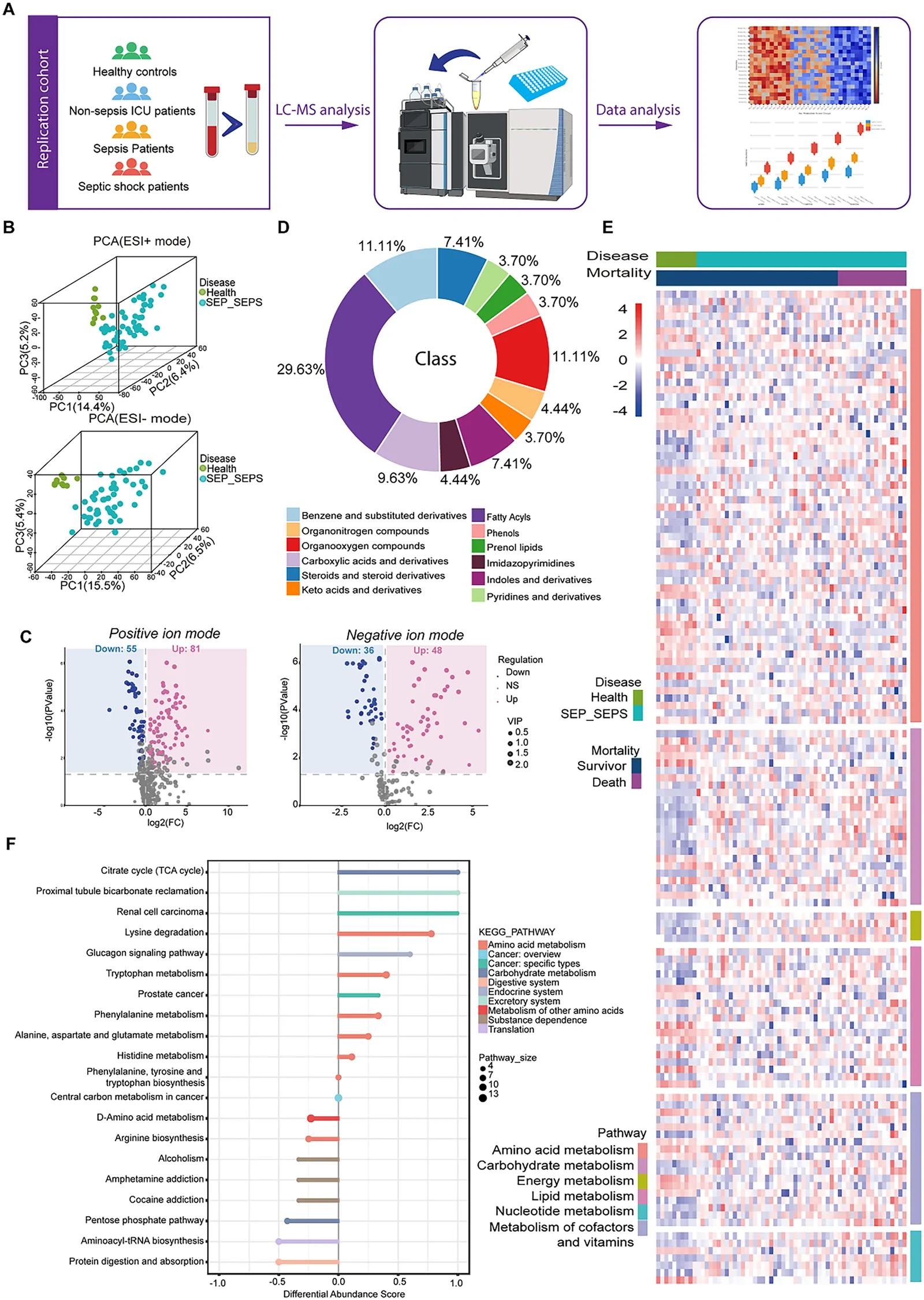
Metabolic profiling reveals significant differences between sepsis patients and healthy controls in the replication cohort. **(A)** Overview of study design and analytical workflow in replication cohort. Flow diagram depicting patient stratification into sepsis, septic shock, ICU non-sepsis, and healthy control groups, followed by standardized blood sample collection, tandem mass spectrometry metabolomic analysis, computational data processing, and comprehensive bioinformatics evaluation. **(B)** 3D PCA plots demonstrating the distinct separation of patient samples between the sepsis group (blue) and the healthy control group (green), indicating significant metabolic differences. **(C)** Volcano plot displaying the statistical significance (log_10_ *p*-value) versus the magnitude of change (log_2_ fold change) for metabolites in the replication cohort. Metabolites with adjusted *p*-values < 0.05 and |log_2_ fold change| > 1 are highlighted, indicating significant differential expression. **(D)** Top 12 categorized pie charts of metabolites. Visual representation of the metabolic differences between sepsis patients and healthy individuals, with each pie chart representing a different metabolic category and the size of each segment indicating the relative abundance of metabolites. **(E)** Hierarchical clustering heatmap of the 131 differential metabolites in the replication cohort (sepsis versus healthy controls), annotated by KEGG metabolic pathway category. Rows represent metabolites and columns represent individual samples (10 healthy controls and 52 sepsis patients); color intensity indicates row-scaled relative abundance. **(F)** Differential abundance score (DA-score) plot of KEGG pathways for the differential metabolites; each point represents a pathway, with the score indicating the overall direction of change (positive = predominantly increased, negative = predominantly decreased) and point size the number of metabolites (pathway size).

### Replication cohort analysis confirms key metabolic pathways and biomarker metabolites for sepsis diagnosis and prognosis

Cross-validation of potential biomarkers between discovery and replication cohorts enabled the identification of robust metabolomic signatures distinguishing sepsis from other conditions. Comparative analysis of differential metabolites across both cohorts revealed 11 metabolites common to the discovery and replication cohorts; of these, nine demonstrated a consistent direction of change (eight upregulated and one downregulated in sepsis) and constituted the candidate metabolite panel (**Figure 5A**); the identification evidence for these metabolites is provided in **Supplementary Table 2**. Heat map analysis of these candidate metabolites demonstrated their capacity to effectively discriminate sepsis patients from both healthy controls and non-sepsis ICU patients in the replication cohort (**Figure 5B**). These metabolites predominantly displayed significantly elevated expression levels in sepsis patients compared with both control groups. Of particular note, N-acetyl-beta-alanine demonstrated higher expression levels in non-survivors compared with survivors, consistent with its elevation in the mortality group. All nine panel features were detected at essentially identical m/z and retention time in both the discovery and replication cohorts, supporting their reproducible identification.

**Figure 5:**
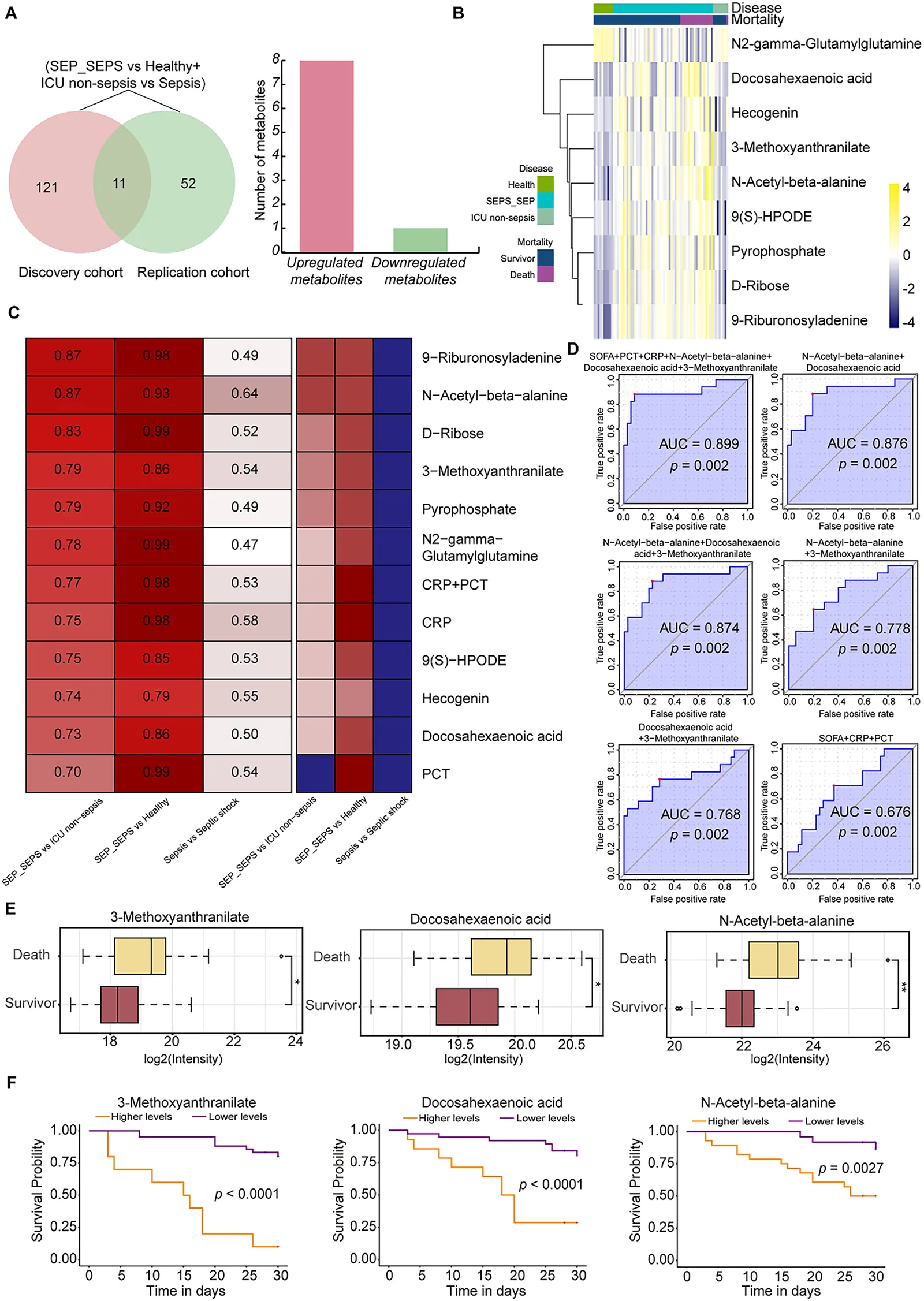
Validation of potential biomarkers in the replication cohort. **(A)** Venn diagram showing the overlap between the significant differential metabolites of the discovery and replication cohorts. Eleven metabolites were common to both cohorts; of these, nine showed a consistent direction of change (eight increased and one decreased in sepsis) and were carried forward as the candidate metabolite panel, while two with discordant direction between cohorts were excluded. **(B)** Heatmap displaying the expression levels of the nine identified differential metabolites in the replication cohort. Rows represent metabolites, columns represent individual samples, and color intensity indicates relative metabolite abundance. **(C)** Receiver operating characteristic (ROC) analysis assessing the diagnostic accuracy of the nine candidate differential metabolites, together with CRP, PCT and CRP+PCT, in distinguishing sepsis patients from healthy controls and non-septic ICU patients in the replication cohort. AUC values are shown for each marker. **(D)** ROC curves assessing the predictive ability of 3-methoxyanthranilate, docosahexaenoic acid and N-acetyl-beta-alanine, individually and in combination, and of the SOFA score with CRP and PCT, for 30-day mortality in the replication cohort. AUC and p-values are shown for each model. The AUC indicates the predictive accuracy for mortality risk. **(E)** Box plot visualizing the expression levels of the top three metabolites in relation to survival and death outcomes in the replication group. **(F)** Kaplan-Meier survival plots illustrating the 30-day mortality rates associated with the top three metabolites in the replication group, comparing survival between patient subgroups.

The diagnostic utility of the nine candidate metabolites was evaluated through ROC curve analyses, showing numerically higher diagnostic accuracy than the combination of CRP and PCT (AUC = 0.77; **Figure 5C**). The metabolite panel effectively discriminated among healthy controls, non-sepsis ICU patients, and sepsis patients. N-acetyl-beta-alanine exhibited enhanced diagnostic efficacy for sepsis compared with conventional biomarkers, despite showing marginally lower discriminatory power between sepsis and septic shock. To evaluate prognostic potential, mortality risk prediction analyses were conducted focusing on three metabolites demonstrating optimal performance: 3-methoxyanthranilate, docosahexaenoic acid, and N-acetyl-beta-alanine. ROC analyses revealed that the combined application of these three metabolites achieved numerically higher predictive accuracy than the SOFA score combined with CRP and PCT (AUC = 0.676; **Figure 5D**). Individual analysis of each metabolite demonstrated enhanced prognostic accuracy compared with the traditional SOFA score combined with CRP and PCT, particularly in terms of specificity. The integration of two or three metabolites further enhanced predictive rates, specificity, and sensitivity. Notably, the combination of these candidate metabolic markers with the clinical SOFA score achieved maximal predictive accuracy and specificity.

To validate these observations, we quantitatively analyzed the expression levels of the three identified metabolites between survival and mortality groups and evaluated their association with 30-day survival outcomes. The concentrations of these metabolites were significantly elevated in the mortality group (*p* < 0.001) and demonstrated strong positive correlations with 30-day mortality rates (r = 0.78, *p* < 0.001), indicating their potential utility as candidate prognostic biomarkers in sepsis (**Figures 5E, F**).

### Temporal dynamics and diagnostic potential of key metabolites in sepsis

To elucidate the temporal dynamics of key metabolites in sepsis progression, we performed longitudinal analyses of 3-methoxyanthranilate, docosahexaenoic acid, and N-acetyl-beta-alanine concentrations in relation to patient outcomes (**Figure 6A**). For the longitudinal analysis, serial serum samples were collected at day 1, day 3 and day 5 from all 23 patients (11 survivors and 12 non-survivors, classified by 30-day outcome), yielding 69 samples with no missing time points. Survivor and non-survivor status refers to 30-day mortality and not to the patient’s condition at the time of sampling. The time-series analysis revealed distinctive temporal trajectories, with 3-methoxyanthranilate and N-acetyl-beta-alanine exhibiting significant divergence by day 5 post-diagnosis between survival and mortality groups (*p* < 0.001). These temporal profiles established robust delineation between outcome groups, highlighting their potential utility as prognostic indicators for monitoring disease progression (**Figure 6B**).

**Figure 6:**
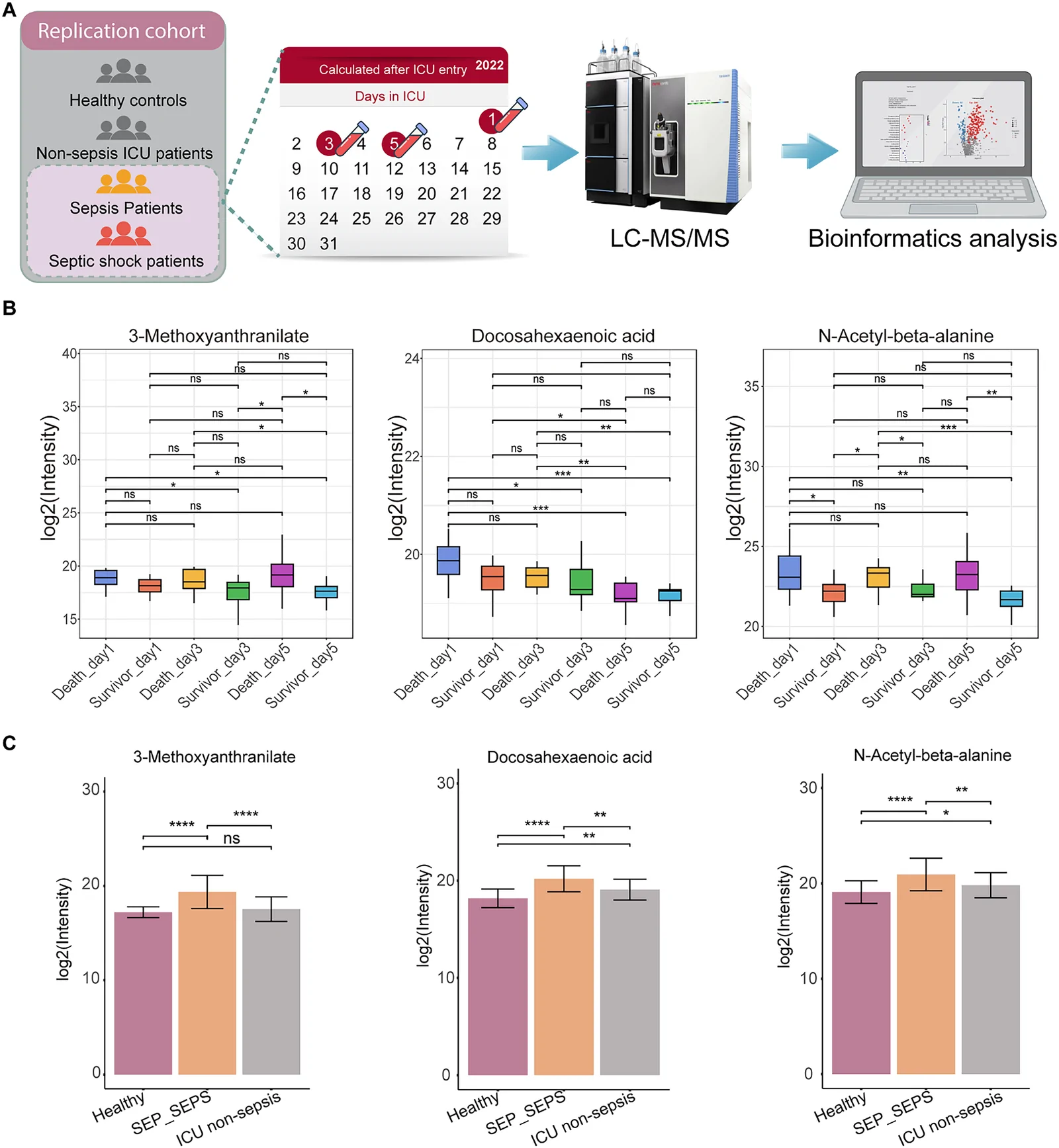
Time-series analysis of metabolic biomarkers in surviving versus dying sepsis patients. **(A)** Serial samples were obtained at day 1, day 3 and day 5 from all 23 longitudinal patients (11 survivors, 12 non-survivors by 30-day outcome; 69 samples in total). Flowchart illustrating the replication cohort, in which blood samples from sepsis and sepsis shock patients were collected on ICU day 1, day 3, and day 5, followed by LC–MS analysis and subsequent bioinformatics processing. **(B)** Box plots displaying the levels of the three identified metabolic biomarkers in both the discovery and replication cohorts at each measured time point (day1, day3, day5). The boxes represent the interquartile range, the horizontal lines within the boxes indicate the median, and the whiskers extend to the minimum and maximum values within 1.5 times the interquartile range. **(C)** Bar charts comparing the levels of the three metabolic biomarkers among three patient cohorts: sepsis patients, non-septic ICU patients, and healthy controls. Bars represent the mean and error bars the standard deviation; statistical significance between groups is indicated.

Furthermore, comparative analysis of metabolite concentrations across three distinct cohorts—sepsis patients, non-sepsis ICU patients, and healthy controls—demonstrated significant inter-group variations (**Figure 6C**). Statistical analyses revealed marked differences in metabolite profiles (ANOVA, *p* < 0.001), substantiating their capacity to discriminate between sepsis and non-sepsis conditions. These findings provide compelling evidence for the diagnostic specificity of these metabolic markers in sepsis identification.

## Discussion

Sepsis represents a complex, life-threatening pathophysiological state characterized by dysregulated inflammatory responses to infection, potentially culminating in multi-organ dysfunction involving pulmonary, hepatic, and renal systems (23, 24). In its most severe manifestation, septic shock develops, characterized by critical impairment of tissue oxygen delivery and nutrient perfusion, leading to profound deterioration in patient outcomes (25, 26). Despite the demonstrated utility of biomarkers in sepsis diagnosis, monitoring, and prognostication, the development of targeted therapeutic interventions remains challenging (27–29). The increasing prevalence of antimicrobial resistance coupled with the decelerated pipeline of novel antibiotic development underscores the critical necessity for innovative approaches in sepsis prevention, diagnosis, and therapeutic intervention (30, 31).

Metabolomics has emerged as an indispensable analytical platform in sepsis research, providing unprecedented insights into the underlying metabolic perturbations. Previous investigations employing metabolomic analyses have revealed significant alterations in metabolic signatures during sepsis progression, facilitating the identification of potential diagnostic and prognostic biomarkers (32, 33). Notably, studies have demonstrated the superior diagnostic utility of whole blood metabolomic profiling compared to plasma analysis, offering a more comprehensive characterization of the metabolic landscape (34–36). Furthermore, metabolomic investigations have contributed substantially to advancing precision medicine approaches in sepsis management by delineating specific biochemical pathways and metabolic signatures integral to sepsis pathophysiology (37–40). The integration of metabolomic profiling with microbiome analysis has expanded the mechanistic understanding of sepsis pathogenesis (41), suggesting the potential of metabolomic approaches for identifying novel biomarkers and therapeutic targets.

Previous metabolomic investigations in sepsis have been constrained by limited sample sizes and the absence of replication cohorts, potentially compromising the robustness and generalizability of their findings. The present study addressed these methodological limitations through implementation of a rigorous dual-cohort design, encompassing both discovery and replication cohorts with a cumulative sample size of 156 subjects. Our comprehensive metabolomic analysis revealed significant perturbations in metabolite profiles among sepsis patients compared with both healthy controls and non-septic ICU patients (*p* < 0.001). The reproducibility of these metabolic alterations across both cohorts strongly suggests their potential utility in enhancing patient outcomes through early detection and targeted therapeutic interventions. A principal contribution of this investigation is the identification and validation of three candidate biomarkers: 3-Methoxyanthranilate (MMA), Docosahexaenoic acid (DHA), and N-Acetyl-beta-alanine (NAA). In this cohort, these metabolites were consistently associated with sepsis diagnosis (area under the curve approximately 0.77 for the candidate panel) and with 30-day mortality and disease progression. Unlike many earlier reports, the candidate markers were examined in a replication cohort; these temporal changes should be interpreted with caution: during ICU stay patients received antibiotics, fluid resuscitation, vasopressors, nutritional support and, in some cases, renal replacement therapy, all of which can substantially alter the plasma metabolome. The observed trajectories therefore cannot be attributed solely to intrinsic metabolic progression of sepsis and may partly reflect these interventions. Nonetheless, the modest single-center sample means these associations remain exploratory and require confirmation in larger studies. Because all annotations were putative (MSI Level 2), targeted assays using authentic reference standards are required to confirm the identity and absolute concentrations of these candidate metabolites—particularly 3-methoxyanthranilate, docosahexaenoic acid and N-acetyl-beta-alanine—before any clinical translation.

This investigation presents the first documented correlation between MMA levels and sepsis progression. While previous studies have implicated tryptophan metabolism in inflammatory cascade regulation (42, 43), the identification of MMA as a specific sepsis biomarker represents a candidate discovery.

DHA, an essential omega-3 fatty acid, exhibits crucial immunomodulatory and anti-inflammatory properties, establishing its significance in inflammatory pathologies (44). The molecular functions of DHA encompass mitochondrial regulation and energy metabolism modulation, processes fundamentally altered in sepsis pathophysiology (45). Our findings corroborate recent evidence suggesting DHA’s protective role in reducing sepsis incidence and associated mortality. Experimental investigations have demonstrated DHA’s protective mechanisms in septic conditions, particularly in attenuating vascular inflammation and oxidative stress (46, 47). The therapeutic potential of DHA in improving clinical outcomes merits systematic investigation through controlled clinical trials. Notably, in this cohort the combination of DHA with MMA and NAA improved diagnostic performance relative to the individual markers (see Results), supporting a multi-marker rather than a single-marker approach.

NAA, an acetylated derivative of beta-alanine, is linked to beta-alanine and pantothenate/CoA-related metabolism. Our findings establish NAA as a prognostic biomarker in sepsis, with significant predictive value for patient survival outcomes. Although NAA has previously been reported to be upregulated in a rat model of CCl4-induced liver fibrosis (48), its biological role in sepsis has not been characterized; the mechanistic basis for its association with mortality therefore remains to be determined and should be regarded as hypothesis-generating. To our knowledge, this study provides the first clinical evidence linking NAA levels to sepsis outcomes.

Our results are broadly consistent with recent metabolomic studies of sepsis. Pandey et al, using NMR-based serum metabolomics, reported sepsis-associated changes in amino-acid and energy-metabolism pathways that overlap with the amino-acid and central-carbon perturbations observed here (49), although their NMR platform and our LC-MS/MS approach differ in metabolite coverage and sensitivity. Chaudhary et al. characterized the serum metabolome in children with sepsis-associated acute kidney injury and highlighted overlapping amino-acid and energy-metabolism signatures (50); the differences from our findings are likely attributable to the pediatric population, the presence of acute kidney injury, and the analytical platform. Taken together, these comparisons support the relevance of amino-acid and energy metabolism in sepsis, while underscoring that candidate biomarker panels are influenced by patient population, organ dysfunction, and analytical method.

The metabolite panel showed numerically higher AUC than CRP and PCT (51), with higher sensitivity and specificity in this cohort (**Figure 5C**). Furthermore, the metabolite panel showed numerically higher prognostic accuracy for mortality prediction than the SOFA score (52) combined with CRP and PCT (**Figure 5D**). Procalcitonin shows limited and variable diagnostic performance across clinical settings and should not be assumed a uniformly reliable comparator, whereas the SOFA score is a well-established prognostic tool in critically ill patients; the metabolite panel is therefore intended to complement, rather than replace, these established markers and scores.

A key consideration is the influence of renal and hepatic dysfunction on the plasma metabolome. Sepsis patients had markedly lower eGFR and higher bilirubin and transaminases than non-septic participants, and the candidate metabolites correlated with renal function, most strongly for 3-methoxyanthranilate (Spearman ρ = −0.70 with eGFR; **Supplementary Figure 8A**; **Supplementary Table 1**). After adjustment for eGFR, total bilirubin, age, sex and cohort, the independent associations of 3-methoxyanthranilate and docosahexaenoic acid with sepsis status were attenuated (**Supplementary Figure 8B**). However, organ dysfunction is intrinsic to sepsis, which Sepsis-3 defines as infection-induced, life-threatening organ dysfunction, so adjusting for these indices over-corrects and removes part of the genuine sepsis-related signal. Reassuringly, among participants with preserved renal function (eGFR ≥ 60 mL/min/1.73 m^2^) all three metabolites remained elevated in sepsis (**Supplementary Figure 8C**), and the mortality associations of 3-methoxyanthranilate and docosahexaenoic acid persisted after eGFR adjustment (**Supplementary Figure 8D**). We therefore cannot fully separate sepsis-specific changes from those secondary to organ dysfunction, and larger cohorts with balanced organ-function strata and baseline renal data are needed. Several methodological limitations warrant consideration in interpreting our findings. Most importantly, the modest, single-center sample size limits the statistical power of the study and the reliability of the candidate biomarkers; the results should therefore be interpreted as exploratory and hypothesis-generating rather than confirmatory. In addition, although baseline renal and hepatic indices were recorded, sepsis-associated organ dysfunction can itself alter the plasma metabolome and may have influenced the candidate metabolites, so residual confounding cannot be excluded and should be examined directly in larger cohorts. First, the single-center design potentially constrains the generalizability of our results across diverse patient populations and clinical settings. Second, the cross-sectional nature of our metabolomic analysis precludes comprehensive characterization of temporal metabolic alterations throughout sepsis progression. Additionally, the implementation of non-targeted metabolomics methodology presents inherent limitations in precisely quantifying differential metabolite concentrations. The identification of MMA, DHA, and NAA as candidate metabolic biomarkers represents a substantial contribution to the field, with numerically higher AUC than conventional markers. Furthermore, formal comparative statistics for the ROC analyses—including 95% confidence intervals, optimal cut-offs with sensitivity, specificity, PPV and NPV, DeLong paired comparisons, model calibration and internal validation—were beyond the scope of this exploratory analysis; the diagnostic and prognostic comparisons reported here should therefore be regarded as exploratory and will be formally evaluated in the planned multi-center validation study. In addition, because the replication cohort was used both to select the final metabolite panel (through the requirement of concordant, cross-cohort differential abundance) and to evaluate its diagnostic and prognostic performance, the reported ROC estimates are not derived from a fully independent sample and may be optimistic; confirmation in an independent external cohort is required.

This investigation has elucidated significant metabolic dysregulations associated with sepsis pathophysiology, culminating in the identification of three candidate metabolic biomarkers: MMA, DHA, and NAA. These metabolites were associated with sepsis diagnosis and 30-day mortality in sepsis patients. Notably, this study presents the first documented association between MMA levels and sepsis progression, representing a significant advancement in biomarker discovery. DHA, an omega-3 fatty acid with established immunomodulatory and anti-inflammatory properties, emerged as a crucial component of the biomarker panel, corroborating previous evidence of its protective effects in septic conditions through attenuation of inflammatory cascades and oxidative stress. The identification of NAA as a prognostic indicator provides candidate insights into sepsis outcomes, although its underlying biology in sepsis remains to be defined.

The metabolite panel showed numerically higher AUC than CRP and PCT, offering enhanced diagnostic accuracy and temporal resolution. Furthermore, the panel’s capacity to differentiate sepsis from other inflammatory conditions in critical care settings addresses a crucial clinical challenge in sepsis diagnosis. This comprehensive investigation establishes a robust foundation for metabolomic applications in sepsis management. The candidate biomarker panel, comprising MMA, DHA, and NAA, shows potential for improving early detection and risk stratification in sepsis, potentially facilitating more targeted therapeutic interventions and improved patient outcomes.

## Data Availability

The datasets presented in this study can be found in online repositories. The names of the repository/repositories and accession number(s) can be found below: ST004550 (DOI: 10.21228/M8PK1B).

## Supplementary material

Supplementary Figure 1 QC analysis of untargeted metabolomics data between sepsis group and healthy control group in the discovery set. **(A)** PCA score plots of QC samples in ESI^+^ and ESI^−^ modes (red: QC; green: Health; blue: SEP_SEPS). **(B)** OPLS-DA score plots of the two groups in ESI^+^ and ESI^−^ modes. **(C)** Permutation tests (n = 200) of the OPLS-DA models in ESI^+^ and ESI^−^ modes (green: R^2^; blue: Q^2^). **(D)** Left: pie chart showing the number of metabolites identified in each ionization mode (ESI^+^: 2,869, 58.1%; ESI^−^: 2,072, 41.9%); right: bar chart showing the number of quantified metabolites with increased (561) versus decreased (180) mean abundance in the sepsis group relative to healthy controls (all 741 quantified metabolites classified by direction of fold change; not filtered by statistical significance).

Supplementary Figure 2 QC analysis of untargeted metabolomics in the sepsis group versus the ICU non-sepsis group in the discovery set. **(A)** PCA score plots of QC samples in ESI^+^ and ESI^−^ modes (red: QC; blue: SEP_SEPS; orange: ICU non-sepsis). **(B)** OPLS-DA score plots of the two groups in ESI^+^ and ESI^−^ modes. **(C)** Permutation tests (n = 200) of the OPLS-DA models in ESI^+^ and ESI^−^ modes (green: R^2^; blue: Q^2^). **(D)** Bar chart showing the number of increased (n = 529) and decreased (n = 212) metabolites in the sepsis group versus the ICU non-sepsis group. (all 741 quantified metabolites classified by direction of fold change; not filtered by statistical significance).

Supplementary Figure 3 Metabolomics analysis identifies significant metabolic differences between sepsis patients and ICU non-sepsis patients in the discovery set. **(A)** 3D PCA showing the separation of patient samples between the sepsis group (blue) and the ICU non-sepsis group (purple), indicating distinct metabolic profiles. **(B)** Volcano plots depicting the statistical significance (log_10_ *p*-value) versus the magnitude of change (log_2_ fold change) for metabolites in the discovery group. Metabolites with adjusted *p*-values < 0.05 and |log2 fold change| > 1 are highlighted. **(C)** Top 12 categorized pie charts of metabolites. Pie charts visually illustrating the metabolic differences between sepsis patients and ICU non-sepsis individuals, with each chart representing a different metabolic category and segment size reflecting relative metabolite abundance. **(D**) Hierarchical clustering heatmap of the 145 differential metabolites in the discovery cohort (sepsis versus non-septic ICU patients), annotated by KEGG metabolic pathway category. Rows represent metabolites and columns represent individual samples (55 sepsis patients and 11 non-septic ICU patients); color intensity indicates row-scaled relative abundance. **(E)** KEGG bar graphs illustrating the distribution of metabolic pathways significantly affected by the differentially expressed metabolites in the discovery group. **(F)** Circular bar plot showing the KEGG pathway enrichment analysis of differential metabolites. Pathways are grouped and color-coded by metabolic category: amino acid metabolism (pink), carbohydrate metabolism (yellow), energy metabolism (green), lipid metabolism (cyan), metabolism of cofactors and vitamins (blue), and nucleotide metabolism (purple). Bar height represents the enrichment level of each pathway, with representative pathways labeled.

Supplementary Figure 4 QC and metabolomic analysis of untargeted metabolomics data between sepsis group and healthy control group in the validation set. **(A)** PCA score plots of QC samples in ESI^+^ and ESI^−^ modes (red: QC; green: Health; blue: SEP_SEPS). **(B)** OPLS-DA score plots of the two groups in ESI^+^ and ESI^−^ modes. **(C)** Permutation tests (n = 200) of the OPLS-DA models in ESI^+^ and ESI^−^ modes (green: R^2^; blue: Q^2^). **(D)** Left: pie chart showing the number of metabolites identified in each ionization mode (ESI^+^: 2,985, 64.6%; ESI^−^: 1,635, 35.4%); right: bar chart showing the number of increased (n = 372) and decreased (n = 260) metabolites in the sepsis group versus the healthy control group (all 632 quantified metabolites classified by direction of fold change; not filtered by statistical significance).

Supplementary Figure 5 QC and metabolomic analysis of untargeted metabolomics data between sepsis group and ICU non-sepsis group in the validation set. **(A)** PCA score plots of QC samples in ESI^+^ and ESI^−^ modes (red: QC; blue: SEP_SEPS; orange: ICU non-sepsis). **(B)** OPLS-DA score plots of the two groups in ESI^+^ and ESI^−^ modes. **(C)** Permutation tests (n = 200) of the OPLS-DA models in ESI^+^ and ESI^−^ modes (green: R^2^; blue: Q^2^). **(D)** Bar chart showing the number of increased (n = 394) and decreased (n = 238) metabolites in the sepsis group versus the ICU non-sepsis group (all 632 quantified metabolites classified by direction of fold change; not filtered by statistical significance).

Supplementary Figure 6 Metabolomics analysis identifies significant metabolic differences between sepsis patients and ICU non-sepsis patients in the validation set **(A)** 3D PCA showing the separation of patient samples between the sepsis group (blue) and the ICU non-sepsis group (purple), indicating distinct metabolic profiles. **(B)** Volcano plots depicting the statistical significance (log_10_ *p*-value) versus the magnitude of change (log_2_ fold change) for metabolites in the replication group. Metabolites with adjusted *p*-values < 0.05 and |log2 fold change| > 1 are highlighted. **(C)** Top 12 categorized pie charts of metabolites. Pie charts visually illustrating the metabolic differences between sepsis patients and ICU non-sepsis individuals, with each chart representing a different metabolic category and segment size reflecting relative metabolite abundance. **(D)** Hierarchical clustering heatmap of the 59 differential metabolites in the replication cohort (sepsis versus non-septic ICU patients), annotated by KEGG metabolic pathway category. Rows represent metabolites and columns represent individual samples (52 sepsis patients and 8 non-septic ICU patients); color intensity indicates row-scaled relative abundance. **(E)** KEGG analysis bubble diagram illustrating the distribution of metabolic pathways significantly affected by the differentially expressed metabolites in the replication group. **(F)** Circular bar plot showing the KEGG pathway enrichment analysis of differential metabolites. Pathways are grouped and color-coded by metabolic category: amino acid metabolism (pink), carbohydrate metabolism (yellow), energy metabolism (green), lipid metabolism (cyan), metabolism of cofactors and vitamins (blue), and nucleotide metabolism (purple). Bar height represents the enrichment level of each pathway, with representative pathways labeled.

Supplementary Figure 7 Screening of potential biomarkers for sepsis diagnosis and prognostic assessment in the replication set. **(A)** Venn diagrams showing the overlap of significantly altered metabolites between comparisons. (Left) Upregulated metabolites in SEP_SEPS compared with healthy controls and ICU non-sepsis patients. (Right) Downregulated metabolites in SEP_SEPS compared with healthy controls and ICU non-sepsis patients. **(B)** Heatmap displaying the expression levels of shared differentially expressed metabolites among sepsis patients (blue), ICU non-sepsis patients (light green) and healthy controls (dark green), with rows representing metabolites and columns representing individual samples. Color intensity reflects the relative metabolite abundance, with higher levels in red and lower levels in green. **(C)** ROC curve assessing the diagnostic efficacy of categorized differentially expressed metabolites in distinguishing sepsis patients from healthy controls. The AUC represents the overall diagnostic accuracy. **(D)** ROC curves evaluating the predictive ability of categorized differentially expressed metabolites for mortality in sepsis patients. The AUC indicates the capacity of these metabolites to forecast the risk of death. **(E)** Bar graph presenting the differential expression levels of representative metabolites in relation to survival (red) and mortality (purple) among sepsis patients, with statistical significance indicated. **(F)** Kaplan-Meier survival plots illustrating the 30-day mortality outcomes associated with the representative metabolites, comparing survival rates between patient groups.

Supplementary Figure 8 Sensitivity analyses accounting for renal and hepatic function. **(A)** Heatmap of Spearman rank-correlation coefficients (ρ) between each metabolite and renal/hepatic indices (eGFR, serum creatinine, and total bilirubin). Color indicates the direction and magnitude of correlation; cell values give ρ with significance (**p* < 0.05, ***p* < 0.01, ****p* < 0.001). All three metabolites correlated inversely with eGFR and positively with creatinine, most strongly for MMA. **(B)** Forest plot of the association between each metabolite and sepsis status (sepsis vs non-sepsis), expressed as OR per 1 SD (95% CI), before adjustment (unadjusted) and after adjustment for eGFR, total bilirubin, age, sex and cohort (adjusted). The vertical dashed line marks OR = 1; the arrow indicates a CI extending beyond the axis limit. After adjustment, the independent associations of MMA and DHA were attenuated and no longer significant; the adjusted estimate for NAA was numerically unstable (very wide CI) and should be interpreted with caution. **(C)** Box plots of log_10_-transformed metabolite abundance in the subgroup with preserved renal function (eGFR ≥ 60 mL/min/1.73 m^2^; 27 sepsis vs 28 non-sepsis), comparing sepsis (red) and non-sepsis (blue). Boxes show the median and interquartile range and whiskers the 1.5× IQR; asterisks denote Mann–Whitney U p-values (***p* < 0.01, ****p* < 0.001). All three metabolites remained significantly elevated in sepsis despite preserved renal function. **(D)** Forest plot of the association between each metabolite and 30-day mortality within the sepsis group, expressed as OR per 1 SD (95% CI), unadjusted and adjusted for eGFR. The associations of MMA and DHA with mortality persisted after adjustment.

Supplementary Table 1 Sensitivity analyses accounting for renal and hepatic function.

Supplementary Table 2 Identification evidence for the nine candidate panel metabolites (discovery cohort; reproducibility confirmed in the replication cohort).

Supplementary Table 3 Differential metabolites with FDR-adjusted *p-*values.

## References

[1] Fleischmann C Scherag A Adhikari NK Hartog CS Tsaganos T Schlattmann P . Assessment of global incidence and mortality of hospital-treated sepsis. Current estimates and limitations. Am J Respir Crit Care Med. (2016) 193:259–72. doi: 10.1164/rccm.201504-0781oc 26414292

[2] Sakr Y Jaschinski U Wittebole X Szakmany T Lipman J Ñamendys-Silva SA . Sepsis in intensive care unit patients: Worldwide data from the intensive care over nations audit. Open Forum Infect Dis. (2018) 5:ofy313. doi: 10.1093/ofid/ofy313 30555852 PMC6289022

[3] de Azambuja Rodrigues PM Valente RH Brunoro GVF Nakaya HTI Araújo-Pereira M Bozza PT . Proteomics reveals disturbances in the immune response and energy metabolism of monocytes from patients with septic shock. Sci Rep. (2021) 11:15149. doi: 10.1038/s41598-021-94474-0 34312428 PMC8313678

[4] Singer M Deutschman CS Seymour CW Shankar-Hari M Annane D Bauer M . The third international consensus definitions for sepsis and septic shock (Sepsis-3). Jama. (2016) 315:801–10. doi: 10.1001/jama.2016.0287 26903338 PMC4968574

[5] Legese MH Asrat D Swedberg G Hasan B Mekasha A Getahun T . Sepsis: emerging pathogens and antimicrobial resistance in Ethiopian referral hospitals. Antimicrob Resist Infect Control. (2022) 11:83. doi: 10.1186/s13756-022-01122-x 35698179 PMC9195281

[6] Goldstein E MacFadden DR Karaca Z Steiner CA Viboud C Lipsitch M . Antimicrobial resistance prevalence, rates of hospitalization with septicemia and rates of mortality with sepsis in adults in different US states. Int J Antimicrob Agents. (2019) 54:23–34. doi: 10.1016/j.ijantimicag.2019.03.004 30851403 PMC6571064

[7] Pandey S . Advances in metabolomics in critically ill patients with sepsis and septic shock. Clin Exp Emerg Med. (2025) 12:4–15. doi: 10.15441/ceem.24.211 PMC12010799 39026452

[8] Gou Y Lv BH Zhang JF Li SM Hei XP Liu JJ . Identifying early predictive and diagnostic biomarkers for patients with sepsis after trauma using untargeted metabolomics. Sci Rep. (2025) 15:12068. doi: 10.1038/s41598-025-92631-3 40199964 PMC11978901

[9] Porru E Comito R Interino N Cerrato A Contoli M Rizzo P . Sulfated bile acids in serum of patients hospitalized with COVID-19. Cells. (2024) 13:1576. doi: 10.3390/cells13181576 PMC11430696 39329758

[10] Corsaro C Vasi S Neri F Mezzasalma AM Neri G Fazio E . NMR in metabolomics: From conventional statistics to machine learning and neural network approaches. Appl Sci. (2022) 12:2824. doi: 10.3390/app1206282430654563

[11] Dettmer K Aronov PA Hammock BD . Mass spectrometry-based metabolomics. Mass Spectrom Rev. (2007) 26:51–78. doi: 10.1002/mas.20108 PMC1904337 16921475

[12] Wu Q Huang Y Kong X Jia B Lu X Chen Y . DBLiPro: A database for lipids and proteins in human lipid metabolism. Phenomics. (2023) 3:350–9. doi: 10.1007/s43657-023-00099-w 37589022 PMC10425311

[13] Letertre MPM Giraudeau P de Tullio P . Nuclear magnetic resonance spectroscopy in clinical metabolomics and personalized medicine: Current challenges and perspectives. Front Mol Biosci. (2021) 8:698337. doi: 10.3389/fmolb.2021.698337 34616770 PMC8488110

[14] Pang H Hu Z . Metabolomics in drug research and development: The recent advances in technologies and applications. Acta Pharm Sin B. (2023) 13:3238–51. doi: 10.1016/j.apsb.2023.05.021 37655318 PMC10465962

[15] Qiu S Cai Y Yao H Lin C Xie Y Tang S . Small molecule metabolites: Discovery of biomarkers and therapeutic targets. Signal Transduction Targeted Ther. (2023) 8:132. doi: 10.1038/s41392-023-01399-3 36941259 PMC10026263

[16] Cai X Xu W Ren C Zhang L Zhang C Liu J . Recent progress in quantitative analysis of self-assembled peptides. Exploration. (2024) 4:20230064. doi: 10.1002/exp.20230064 39175887 PMC11335468

[17] Ahn WG Jung JS Kwon HY Song DK . Alteration of lysophosphatidylcholine-related metabolic parameters in the plasma of mice with experimental sepsis. Inflammation. (2017) 40:537–45. doi: 10.1007/s10753-016-0499-8 28028754

[18] Chen W Guo W Li Y Chen M . Integrative analysis of metabolomics and transcriptomics to uncover biomarkers in sepsis. Sci Rep. (2024) 14:9676. doi: 10.1038/s41598-024-59400-0 38678059 PMC11055861

[19] Zelena E Dunn WB Broadhurst D Francis-McIntyre S Carroll KM Begley P . Development of a robust and repeatable UPLC-MS method for the long-term metabolomic study of human serum. Anal Chem. (2009) 81:1357–64. doi: 10.1021/ac8019366 19170513

[20] Want EJ Masson P Michopoulos F Wilson ID Theodoridis G Plumb RS . Global metabolic profiling of animal and human tissues via UPLC-MS. Nat Protoc. (2013) 8:17–32. doi: 10.1038/nprot.2012.135 23222455

[21] Agerbo Rasmussen J Rømer Villumsen K Ernst M Hansen M Forberg T Gopalakrishnan S . A multi-omics approach unravels metagenomic and metabolic alterations of a probiotic and synbiotic additive in rainbow trout (*Oncorhynchus mykiss*). Microbiome. (2022) 10(1):21. doi: 10.1186/s40168-021-01221-8 PMC8802455 35094708

[22] Costa C Maraschin M Rocha M . An R package for the integrated analysis of metabolomics and spectral data. Comput Methods Programs BioMed. (2016) 129:117–24. doi: 10.1016/j.cmpb.2016.01.008 26853041

[23] Jarczak D Kluge S Nierhaus A . Sepsis-pathophysiology and therapeutic concepts. Front Med (Lausanne). (2021) 8:628302. doi: 10.3389/fmed.2021.628302 34055825 PMC8160230

[24] Sun H Xiao L . Overview of the epidemic characteristics of Mycoplasma pneumoniae infection around COVID pandemic. iLABMED. (2023) 1:148–57. doi: 10.1002/ila2.2742587375

[25] Hotchkiss RS Moldawer LL Opal SM Reinhart K Turnbull IR Vincent JL . Sepsis and septic shock. Nat Rev Dis Primers. (2016) 2:16045. doi: 10.1038/nrdp.2016.45 28117397 PMC5538252

[26] Yu R Chen A Zhang Y . SARS-CoV-2: Possible role in children with acute hepatitis of unknown origin. hLife. (2023) 1:83–92. doi: 10.1016/j.hlife.2023.10.008 42903466 PMC13681516

[27] Jang JH Choi E Kim T Yeo HJ Jeon D Kim YS . Navigating the modern landscape of sepsis: Advances in diagnosis and treatment. Int J Mol Sci. (2024) 25(13):7396. doi: 10.3390/ijms25137396 39000503 PMC11242529

[28] Zhou F Chen M Liu Y Xia X Zhao P . Serum mitochondrial-encoded NADH dehydrogenase 6 and Annexin A1 as novel biomarkers for mortality prediction in critically ill patients with sepsis. Front Immunol. (2024) 15:1486322. doi: 10.3389/fimmu.2024.1486322 39611143 PMC11602424

[29] Chen J Liu Z Zhou F Sun Y Jiang Z Zhao P . Assessment of S100A8/A9 and resistin as predictive biomarkers for mortality in critically ill patients with sepsis. Front Cell Infect Microbiol. (2025) 15:1555307. doi: 10.3389/fcimb.2025.1555307 40568710 PMC12188459

[30] Jackson KE Semler MW . Advances in sepsis care. Clin Chest Med. (2022) 43:489–98. doi: 10.1016/j.ccm.2022.05.003 36116816 PMC10284258

[31] Pant A Mackraj I Govender T . Advances in sepsis diagnosis and management: A paradigm shift towards nanotechnology. J BioMed Sci. (2021) 28:6. doi: 10.1186/s12929-020-00702-6 33413364 PMC7790597

[32] Standage SW Xu S Brown L Ma Q Koterba A Lahni P . NMR-based serum and urine metabolomic profile reveals suppression of mitochondrial pathways in experimental sepsis-associated acute kidney injury. Am J Physiol Renal Physiol. (2021) 320:F984–F1000. doi: 10.1152/ajprenal.00582.2020 33843271 PMC8424559

[33] Fanos V Stronati M Gazzolo D Corsello G . Metabolomics in the diagnosis of sepsis. Ital J Pediatr. (2014) 40:A11. doi: 10.1186/1824-7288-40-s1-a1138164791

[34] Stringer KA Younger JG McHugh C Yeomans L Finkel MA Puskarich MA . Whole blood reveals more metabolic detail of the human metabolome than serum as measured by 1H-NMR spectroscopy: Implications for sepsis metabolomics. Shock. (2015) 44:200–8. doi: 10.1097/shk.0000000000000406 26009817 PMC4537695

[35] Kauppi AM Edin A Ziegler I Mölling P Sjöstedt A Gylfe Å . Metabolites in blood for prediction of bacteremic sepsis in the emergency room. PLoS One. (2016) 11:e0147670. doi: 10.1371/journal.pone.0147670 26800189 PMC4723089

[36] Liu S Huang Q Zhang X Dong W Zhang W Wu S . Cardiorespiratory effects of indoor ozone exposure associated with changes in metabolic profiles among children: A repeated-measure panel study. Innovation. (2021) 2(1):100087. doi: 10.1016/j.xinn.2021.100087 34557741 PMC8454695

[37] Eckerle M Ambroggio L Puskarich MA Winston B Jones AE Standiford TJ . Metabolomics as a driver in advancing precision medicine in sepsis. Pharmacotherapy. (2017) 37:1023–32. doi: 10.1002/phar.1974 28632924 PMC5600684

[38] Evangelatos N Bauer P Reumann M Satyamoorthy K Lehrach H Brand A . Metabolomics in sepsis and its impact on public health. Public Health Genomics. (2017) 20:274–85. doi: 10.1159/000486362 29353273

[39] Pan T Sun S Chen Y Tian R Chen E Tan R . Immune effects of PI3K/Akt/HIF-1α-regulated glycolysis in polymorphonuclear neutrophils during sepsis. Crit Care. (2022) 26:29. doi: 10.1186/s13054-022-03893-6 35090526 PMC8796568

[40] She H Hu Y Zhou Y Tan L Zhu Y Ma C . Protective effects of dexmedetomidine on sepsis-induced vascular leakage by alleviating ferroptosis via regulating metabolic reprogramming. J Inflammation Res. (2021) 14:6765–82. doi: 10.2147/jir.s340420 34916824 PMC8670891

[41] Lee J Banerjee D . Metabolomics and the microbiome as biomarkers in sepsis. Crit Care Clin. (2020) 36:105–13. doi: 10.1016/j.ccc.2019.08.008 31733672

[42] Sorgdrager FJH Naudé PJW Kema IP Nollen EA Deyn PP . Tryptophan metabolism in inflammaging: From biomarker to therapeutic target. Front Immunol. (2019) 10:2565. doi: 10.3389/fimmu.2019.02565 31736978 PMC6833926

[43] Seo S-K Kwon B . Immune regulation through tryptophan metabolism. Exp Mol Med. (2023) 55:1371–9. doi: 10.1038/s12276-023-01028-7 37394584 PMC10394086

[44] Abdul H Laishram S . DHA (Docosahexaenoic Acid): A biomolecule with diverse roles and health benefits. In: Rao AV Leticia R , editors. Dietary Supplements. IntechOpen, Rijeka (2023). p. Ch. 4. doi: 10.5772/intechopen.109677

[45] Lei P Xu W Wang C Lin G Yu S Guo Y . Mendelian randomization analysis reveals causal associations of polyunsaturated fatty acids with sepsis and mortality risk. Infect Dis Ther. (2023) 12:1797–808. doi: 10.1007/s40121-023-00831-z 37316614 PMC10390410

[46] López-Alarcón M Bernabe-García M Del Prado M Rivera D Ruiz G Maldonado J . Docosahexaenoic acid administered in the acute phase protects the nutritional status of septic neonates. Nutrition. (2006) 22:731–7. doi: 10.1016/j.nut.2006.03.008 16750345

[47] Wang C Han D Feng X Hu L Wu J . Docosahexaenoic acid alleviates LPS-induced cytotoxicity in HL-1 cardiac cells via improving stress-induced mitochondrial fragmentation. Heliyon. (2023) 9:e22465. doi: 10.1016/j.heliyon.2023.e22465 38107281 PMC10724566

[48] Gao J Qin XJ Jiang H Chen JF Wang T Zhang T . Detecting serum and urine metabolic profile changes of CCl4-liver fibrosis in rats at 12 weeks based on gas chromatography-mass spectrometry. Exp Ther Med. (2017) 14(2):1496–504. doi: 10.3892/etm.2017.4668 28810615 PMC5525970

[49] Pandey S Azim A Sinha N . NMR-based serum metabolomics in the diagnosis of sepsis and septic shock. Biophys Chem. (2025) 326:107491. doi: 10.1016/j.bpc.2025.107491 40680600

[50] Chaudhary N Singh P Saha A Sengupta S . Serum metabolomic profile in children with sepsis-associated acute kidney injury. Clin Exp Pediatr. (2026) 69:545–54. doi: 10.3345/cep.2025.02439 41986953 PMC13337310

[51] Cui N Zhang H Chen Z Yu Z . Prognostic significance of PCT and CRP evaluation for adult ICU patients with sepsis and septic shock: retrospective analysis of 59 cases. J Int Med Res. (2019) 47:1573–9. doi: 10.1177/0300060518822404 30656987 PMC6460616

[52] Vincent JL de Mendonça A Cantraine F Moreno R Takala J Suter PM . Use of the SOFA score to assess the incidence of organ dysfunction/failure in intensive care units: results of a multicenter, prospective study. Working group on "sepsis-related problems" of the European Society of Intensive Care Medicine. Crit Care Med. (1998) 26:1793–800. doi: 10.1097/00003246-199811000-00016 9824069

