# Supplementary material for: Plasma metabolomic profiling identifies candidate biomarkers associated with sepsis diagnosis and mortality prediction
Source: Front Immunol. 2026 Oct 14;17:1931808. doi: 10.3389/fimmu.2026.1931808 (PMC13713347; doi:10.3389/fimmu.2026.1931808)
Supplement: Supplementary file 1 [file Table1.docx]

**Supplementary Table 1. Sensitivity analyses accounting for renal and hepatic function.**

| **Analysis** | **MMA** | **DHA** | **NAA** |
| --- | --- | --- | --- |
| **Spearman correlation with organ function [ρ; *p*]** | | | |
| eGFR | -0.70; *p* = <0.001 | -0.45; *p* = <0.001 | -0.39; *p* = <0.001 |
| Creatinine | +0.67; *p* = <0.001 | +0.43; *p* = <0.001 | +0.37; *p* = <0.001 |
| Total bilirubin | +0.29; *p* = 0.001 | +0.30; *p* = <0.001 | +0.02; *p* = 0.814 |
| **Sepsis vs non-sepsis — OR per 1 SD (95% CI); *p*** | | | |
| Unadjusted | 10.07 (4.36–23.26); *p* = <0.001 | 7.50 (3.66–15.38); *p* = <0.001 | 3.35 (2.04–5.49); *p* = <0.001 |
| Adjusted† | 2.29 (0.85–6.16); *p* = 0.100 | 1.45 (0.48–4.42); *p* = 0.511 | 29.02 (2.99–281.67); *p* = 0.004 |
| **Preserved renal function (eGFR ≥ 60; 27 sepsis vs 28 non-sepsis)** | | | |
| Sepsis vs non-sepsis | higher in sepsis; *p* = 0.002 | higher in sepsis; *p* = <0.001 | higher in sepsis; *p* = 0.002 |
| **30-day mortality within sepsis — OR per 1 SD (95% CI); p** | | | |
| Unadjusted | 2.12 (1.35–3.35); *p* = 0.001 | 2.22 (1.37–3.61); *p* = 0.001 | 1.50 (0.99–2.28); *p* = 0.053 |
| Adjusted for eGFR | 2.21 (1.31–3.72); *p* = 0.003 | 2.14 (1.29–3.55); *p* = 0.003 | 1.45 (0.94–2.22); *p* = 0.093 |
